# Supplementary material for: Upskilling health and care workers with augmented and virtual reality: protocol for a realist review to develop an evidence-informed programme theory
Source: BMJ Open. 2021 Jul 5;11(7):e050033. doi: 10.1136/bmjopen-2021-050033 (PMC8258595; doi:10.1136/bmjopen-2021-050033)
Supplement: Author's manuscript [file bmjopen-2021-050033.draft_revisions.pdf]

BMJ Open is committed to open peer review. As part of this commitment we make the peer review history of every article we publish publicly available.

When an article is published we post the peer reviewers' comments and the authors' responses online. We also post the versions of the paper that were used during peer review. These are the versions that the peer review comments apply to.

The versions of the paper that follow are the versions that were submitted during the peer review process. They are not the versions of record or the final published versions. They should not be cited or distributed as the published version of this manuscript.

BMJ Open is an open access journal and the full, final, typeset and author-corrected version of record of the manuscript is available on our site with no access controls, subscription charges or pay-per-view fees (http://bmjopen.bmj.com).

If you have any questions on BMJ Open's open peer review process please email info.bmjopen@bmj.com

BMJ Open

Upskilling health and care workers with augmented and virtual reality: Protocol for a realist review to develop an evidence-informed programme theory

Journal:	*BMJ Open*
Manuscript ID	bmjopen-2021-050033
Article Type:	Protocol
Date Submitted by the Author:	08-Feb-2021

Complete List of Authors:	Gasteiger, Norina; The University of Manchester Faculty of Biology Medicine and Health, Division of Nursing, Midwifery & Social Work; The University of Manchester Faculty of Biology Medicine and Health, Centre for Health Informatics, Division of Informatics, Imaging and Data Sciences<br>van der Veer, Sabine N.; The University of Manchester Faculty of Biology Medicine and Health, Centre for Health Informatics, Division of Informatics, Imaging and Data Sciences; The University of Manchester, Manchester Academic Health Science Centre<br>Wilson, Paul; The University of Manchester Faculty of Biology Medicine and Health, Division of Population Health, Health Services Research & Primary Care<br>Dowding, Dawn; The University of Manchester Faculty of Biology Medicine and Health, Division of Nursing, Midwifery & Social Work

Keywords:	MEDICAL EDUCATION & TRAINING, EDUCATION & TRAINING (see Medical Education & Training), Information technology < BIOTECHNOLOGY & BIOINFORMATICS, Health informatics < BIOTECHNOLOGY & BIOINFORMATICS

SCHOLARONE™
Manuscripts

I, the Submitting Author has the right to grant and does grant on behalf of all authors of the Work (as defined in the below author licence), an exclusive licence and/or a non-exclusive licence for contributions from authors who are: i) UK Crown employees; ii) where BMJ has agreed a CC-BY licence shall apply, and/or iii) in accordance with the terms applicable for US Federal Government officers or employees acting as part of their official duties; on a worldwide, perpetual, irrevocable, royalty-free basis to BMJ Publishing Group Ltd ("BMJ") its licensees and where the relevant Journal is co-owned by BMJ to the co-owners of the Journal, to publish the Work in this journal and any other BMJ products and to exploit all rights, as set out in our licence.

The Submitting Author accepts and understands that any supply made under these terms is made by BMJ to the Submitting Author unless you are acting as an employee on behalf of your employer or a postgraduate student of an affiliated institution which is paying any applicable article publishing charge ("APC") for Open Access articles. Where the Submitting Author wishes to make the Work available on an Open Access basis (and intends to pay the relevant APC), the terms of reuse of such Open Access shall be governed by a Creative Commons licence – details of these licences and which Creative Commons licence will apply to this Work are set out in our licence referred to above.

Other than as permitted in any relevant BMJ Author's Self Archiving Policies, I confirm this Work has not been accepted for publication elsewhere, is not being considered for publication elsewhere and does not duplicate material already published. I confirm all authors consent to publication of this Work and authorise the granting of this licence.

For peer review only

Upskilling health and care workers with augmented and virtual reality: Protocol for a realist review to develop an evidence-informed programme theory

Norina Gasteiger[1,2,3,*], Sabine N van der Veer[2,4], Paul Wilson[3] and Dawn Dowding[1]

1. Division of Nursing, Midwifery & Social Work, University of Manchester, Manchester, UK

2. Centre for Health Informatics, Division of Informatics, Imaging and Data Sciences, University of Manchester, Manchester, UK

3. Division of Population Health, Health Services Research & Primary Care, University of Manchester, Manchester, UK

4. Manchester Academic Health Science Centre, University of Manchester, Manchester, UK

***Corresponding author:**
Norina Gasteiger
Location: Jean McFarlane Building, Oxford Road, Manchester, M13 9PL, United Kingdom
Email: norina.gasteiger@postgrad.manchester.ac.uk

ORCiD:
N. Gasteiger: 0000-0001-7801-7417
S.N van der Veer: 0000-0003-0929-436X
P. Wilson: 0000-0002-2657-5780
D. Dowding: 0000-0001-5672-8605

For peer review only

ABSTRACT

Introduction

Augmented reality (AR) and virtual reality (VR) are increasingly used to upskill health and care providers, including in surgical, nursing and acute care settings. Many studies have used AR/VR to deliver training, providing mixed evidence on their effectiveness and limited evidence regarding contextual factors that influence effectiveness and implementation. This review will develop, test and refine an evidence-informed programme theory on what facilitates or constrains the implementation of AR or VR programmes in health and care settings and understand how, for whom and to what extent they 'work.'

Method and analysis

This realist review adheres to the RAMESES standards and will be conducted in three steps: theory elicitation, theory testing, and theory refinement. First, a search will identify practitioner, academic and learning and technology adoption theories from databases (Medline, SCOPUS, CINAHL, EMBASE, Education Resource Information Centre, PsycINFO and Web of Science), practitioner journals, snowballing and grey literature. Information regarding context, mechanism and outcome will be extracted. A narrative synthesis will determine overlapping configurations and form an initial theory. Second, the theory will be tested using empirical evidence located from the above databases. Quality will be assessed using the Mixed Methods Appraisal Tool (MMAT), and relevant information will be extracted into a coding sheet. Third, the extracted information will be compared to the initial programme theory, with differences helping to make refinements. Findings will be presented as a narrative summary, and the MMAT will determine our confidence in each configuration.

Ethics and dissemination

Ethics approval is not required. This review will develop an evidence-informed programme theory. The results will inform and support AR/VR interventions from clinical educators, healthcare providers and software developers. Upskilling through AR/VR learning interventions may improve quality of care and promote evidence-based practice and continued learning. Findings will be disseminated through conference presentations and peer-reviewed journal articles.

KEYWORDS

healthcare; learning; simulation; virtual reality; augmented reality; realist review

STRENGTHS AND LIMITATIONS OF THIS STUDY

- The realist approach helps to understand the contextual factors that shape the implementation and impact of AR/VR interventions to upskill professionals in health and care settings.
- The programme theory may be theoretically transferable, as it includes literature from various health and care contexts.
- The inclusion of the MMAT will help stakeholders use the theory by stating the extent to which we are confident in each configuration.
- This realist review focuses on AR and VR, so may need to be modified to apply to other digital simulation technologies.
- Literature included in this review is limited to that published in English.

WORD COUNT: 3,990

For peer review only

INTRODUCTION

Upskilling in the health and care workforce

Upskilling through continuous learning and development is important in any business, to improve skillsets, advance practice and close gaps in knowledge. Upskilling is the process of learning new skills or refining existing skillsets to enable employees to continue practising with ease[1]. For health support and care workers, upskilling ensures that their work is safe and aligns with best practice guidelines, as they often receive variable and inconsistent training, as non-registered staff[2, 3]. Upskilling, in this sense, is therefore essential for providing consistent and high-quality care. Additionally, this promotes workforce flexibility and enables for the delegation of skills, when systems experience a shortage of staff[4]. Within the provision of health and care, upskilling is also crucial when adapting in times of change[5, 6] or crisis[7] and to align with up-to-date best practice.

Health and care providers may range from registered clinicians such as surgeons, general practitioners and doctors, nurses and midwives, to allied health and non-registered staff who provide care. Allied health staff may include paramedics, dieticians, podiatrists and radiographers[8], while carers also include those working for care-based organisations such as in care homes or homecare agencies. Regardless of the role, all staff that provide health and care services must act in accordance with policies/guidelines and optimally engage in up-to-date evidence-based best practice.

Evidence-based practice is widely recognised as the gold standard when providing effective and safe healthcare[9]. This requires professionals to update and upskill themselves on current evidence and to alter their practice to align with this, as well as with their patient's preferences[10]. Current evidence is usually retrieved from peer-reviewed journal articles; however, due to time constraints and workload demand, many health and care staff rely on organisational policies and protocols as formal sources of knowledge[11]. As the evidence base grows, old habits must be adapted and upskilling is required to align with the newest best practice.

Upskilling is also essential when adapting in times of change or crisis. For example, the emergence of medical and healthcare technologies requires staff to upskill, including improving their digital literacy skills[5, 6]. Additionally, the novel coronavirus disease-19 (COVID-19) pandemic caused significant changes to health and care systems. Changes included staff deployment to wards (e.g. COVID-19 wards) outside of their normal experience and of retired and newly qualified staff, remote provision of healthcare using telehealth (phones, video, patient portals), distancing/minimal contact care, stringent use of personal protective equipment and strengthened inter-professional collaboration[12-15]. These challenges required prompt upskilling, especially in using technologies and in infection, prevention and control behaviours to minimise the spread of COVID-19.

Upskilling training programmes

Upskilling training programmes traditionally consist of e-learning, textbooks, workshops, seminars, shadowing/observation and reading peer-reviewed journal articles. Hatfield et al.[16] systematically reviewed 12 studies that delivered behaviour change training interventions to healthcare professionals. All used educational elements (e.g. presentations and workshops) and most were delivered in-person. Morris et al.[17] reviewed training interventions aimed at carers. Both reviews concluded that interventions that use both educational and practical elements (e.g. practising skills or discussion) are

For peer review only

most effective [16, 17]. This indicates that education-only interventions may not be effective in upskilling health and care staff.

Time, organisational structure, difficult to access resources and a reliance on experiential knowledge also constrain providers from upskilling[3, 11]. Health and care staff have widely reported a preference for learning through ‘doing’ (such as interacting with or observing colleagues), rather than from journal articles or textbooks[18-20]. Additionally, although support and care workers provide clinical, care-based and clerical patient care, their value is often not reflected in their allocated training budgets and available programmes[3]. As a result, many feel insufficiently prepared[3]. However, clinical, health support and care staff indicate a willingness to upskill, receive further training and to participate in interventions that will improve their practice[11, 21]. Further, some managers and nurses in England-based nursing homes have expressed enthusiasm toward implementing innovative digital health technologies that may improve residents' quality of care[22].

Digital technologies for upskilling

Effective interventions that are short, accessible, interactive, memorable and low cost are needed, to overcome training barriers. For workplaces with staff shortages, training also needs to be flexible and provided on a drop-by basis[3]. Brief interventions delivered via digital technology may be appropriate, as they can be made available online and accessed 24/7. They can also be more engaging and memorable, by including interactive activities (e.g. games, quizzes, simulations and immediate performance feedback). However, there is limited literature on implementation strategies for digital interventions that upskill health and care workers. Theories of change can be applied to knowledge of existing barriers and facilitators to using digital health programmes for healthcare workers. Lewin describes behaviour as “a dynamic balance of forces working in opposing directions” [23]. Lewin theorises that driving forces (i.e. facilitators) and restraining forces (i.e. barriers) counter one another, but can result in change if one overrides the other. This means that barriers and facilitators directly impact the implementation success and effectiveness of digital training programmes for health and care staff.

Literature on digital health technologies has highlighted various driving and restraining forces that impact both implementation and the effectiveness of programmes. Keyworth et al.[24] conducted a review of 69 studies to determine what maximises the effectiveness and implementation of technology-based interventions that support healthcare professional practice. They concluded that successful technologies employ behaviour change theories and specific instruction on how to perform behaviours. They also provide professionals with knowledge and person-specific information to assist with practice (e.g. patient management). Driving forces for implementation include integration into clinical workload, alignment with organisational strategies and senior peer endorsement. Restraining forces include organisational challenges, as well as the design, content and technical issues of the interventions.

Literature also highlights key strategies for implementation, focussing on provider adoption and acceptance. Recommendations for facilitating change include linking new practice with old practice to build familiarity[25, 26], identifying people who are willing to facilitate and promote the new practice[26, 27] and to clearly communicate to staff as to how the new practice will benefit them and their patients[26, 28, 29]. Spagnoletti et al.[28] provide specific examples, highlighting that short sessions, role-modelling content (e.g. video clips of the behaviour) and modules that refresh

For peer review only

understanding of familiar curriculum were important in their implementation of an online training programme for interns.

Simulation technologies for upskilling

The implementation of simulation technologies may be a novel and engaging approach to upskilling health and care workers. Simulation in this context refers to the replication of real-life interactions or scenarios, whereby learners receive immediate feedback/de-briefing[30]. Various levels of simulation exist, depending on 'fidelity' (reality). According to Seropian et al.[31], these can be categorised as high, medium and low fidelity and use tools such as human-like body parts, haptic feedback, computer programmes (e.g. serious games) or virtual reality (VR) headsets to facilitate experimental learning. Low fidelity simulation may include a simple body part, such as a doll-like arm to practice intravenous insertion skills[32]. In contrast, high fidelity simulation tools include real-life responses driven by computers[32]. These are more expensive and may include the METI Human Patient Simulator, which looks and acts like a human (e.g. blinks, has a pulse and speaks) and accurately mirrors responses to clinical procedures, such as intubation and catheterisation. However, it is important to note that simulators mimic, rather than replicate reality[32].

Simulation technology has been found to be as effective as traditional teaching methods for educating health and care staff and students[33-35]. However, when compared to traditional methods, students report better retention of knowledge[36] and higher satisfaction and motivation when using simulation technologies such as games[34]. Experimental learning by simulation also allows for learners to repeatedly practice skills and make and learn from their mistakes without harming a patient, distressing them or facing other negative consequences[32, 37]. Computer-driven simulation technologies such as games, augmentation and VR also enable independent learning, without the need for an instructor to provide feedback or de-brief learners.

In VR, users wear a headset to become immersed in a digital environment. Headsets range from the low-cost Samsung Gear VR or Google Cardboard to high-end gaming equipment such as Oculus Touch. The extent of immersion also differs, ranging from non-immersion (e.g. using computer-based VR), semi-immersion and fully immersive simulations (e.g. those with haptic feedback). The perception of being immersed within a non-physical world is created through various stimuli, including images and sound[38], which enable users to learn from experience. In interactive medical VR, users can engage in virtual worlds, including with patients, colleagues and react to specific scenarios[30]. In contrast, within augmented reality (AR) real-world environments are complemented with interactive computer-generated imagery and information.

Unlike traditional simulators, the main benefit of VR is transporting the learner into an immersive environment, rather than an educational one. VR and AR interventions are also cost-effective as they can be used autonomously, independently, and repeatedly, compared to traditional simulation technologies. In fact, they have been deemed as the learning tool of the 21st century[39] and their popularity is expected to continually increase. Current projections for the AR/VR head-mounted display market include a worth of USD 25 billion by 2022, with an annual growth rate of 39.5%[40]. This highlights that now is the ideal time to research implementation of AR/VR, due to an inevitable growth in use and further reduction in costs.

These technologies have transformed clinical training and have been used to support health care workers in decision-making and teaching emergency response, resuscitation, robotic surgery and

For peer review only

alcohol screening skills[41-45]. However, their effectiveness is contested within the literature, with some research stating that VR is not as effective as other training tools, including for phlebotomy training[46]. Other literature highlights that VR is useful for 'presence', but does not improve learning outcomes[47, 48]. It is hypothesised that VR increases cognitive load and therefore compromises cognitive resources from the learning experience[47]. Conversely, some research has found VR to be more effective than other educational techniques[49, 50], with systematic reviews concluding that VR training is effective in improving technical skills for arthroscopic surgery[51] and knowledge and skill performance when learning clinical psychomotor skills[52]. Evidently, research is needed to explore to what extent and for whom VR interventions are effective.

Despite their contested effectiveness, VR and AR technologies have now been commercialised and implemented to upskill and support health providers. FundamentalVR[53], for example, provides flight simulator-like training for surgeons with the use of haptic elements for tactile feedback. In the SentiAR[54] tool, holographic visualisations are provided for each patients anatomy and float alongside or above the patient during procedures (e.g. treating cardiac arrhythmias). Other tools include the AR xVision[55] three-dimensional anatomical images that enable clinical providers to see a patient's skin and tissue (akin to x-ray vision) and the AR SureWash[56] mobile app, which provides personalised feedback for hand hygiene technique. VR technologies were also implemented during the COVID-19 pandemic when face-to-face teaching was not possible[57]. For example, St Bartholomew's Hospital used VR to train their nurses and doctors on 50 clinical procedures[58]. Their OMS VR system provided performance feedback, tracked improvement and facilitated group learning.

Gap in research and aim

Despite the emergence and potential effectiveness of simulation technologies, there is an absence of research on their effectiveness as an educational intervention. This includes how good they are at enabling upskilling compared to other strategies, and how they can be implemented into a practice setting, to enable upskilling. Additionally, as evident in the mixed findings on the effectiveness of AR and VR interventions in upskilling staff, programme interventions, including digital ones, do not work for everyone equally[59]. A gap in research remains on the factors that influence when an AR or VR intervention works, to what extent, for whom and in which context. Moreover, research is needed on the causal mechanisms that influence the outcomes of AR/VR interventions and their implementation. This is essential in ensuring that future digital interventions are designed and appropriately targeted at health and care workers, for both maximum efficiency and sustained effects. The aim of this review is to develop, test and refine an evidence-informed programme theory on what facilitates or constrains the implementation of AR or VR programmes in health and care settings and understand how, for whom and to what extent they 'work.'

METHODS AND ANALYSIS

Realist review

This research will take a realist approach because it can produce useful answers to complex questions often left unexplored by experimental research[59]. These questions include: how, when, for whom and to what extent, does an intervention 'work'? To answer these questions, realist approaches consider the complex interactions between the environment, individuals and the intervention.

For peer review only

Realist evaluation is an emerging theory-driven methodology that seeks to understand CMO configurations, i.e. the context (C), mechanisms (M) and outcomes (O) of interventions. Context refers to the backdrop of conditions that may impact outcomes, such as organisational structure, environmental settings, culture and norms. These trigger or modify mechanisms (causal forces) that influence outcomes[60]. Examples of mechanisms include the resources offered by interventions or changes in reasoning or behaviour.

Realist reviews seek to understand context, mechanisms and outcomes, by identifying candidate theories and then systematically reviewing literature for underlying social entities, processes or social structures that result in the intended outcome[61]; rather than assuming that the intervention itself produces an outcome. This process is useful for complex interventions, in which outcomes may not necessarily be linear, and instead depend on the context and both intended and unintentional mechanisms[61]. It also allows exploring how an intervention is meant to work compared to how it actually works in practice[62]. Additionally, 'demi-regularities' are identified to acknowledge that outcomes will vary across contexts, but some CMO patterns will remain[60]. This focuses reviewers on the transferable aspects of a programme theory[61].

CMO configurations are then developed as a programme theory, which is tested and refined in real-life settings and with key stakeholders[59]. As with AR/VR technologies, the main benefit of realist evaluation is the ability to bridge theory and practical application in the contexts and with the populations that the intervention targets[59].

A realist review will therefore help to answer the following questions:

- What facilitates or constrains the implementation of AR/VR programmes in health and care settings?
- What are the mechanisms by which VR/AR interventions result in their intended outcomes?
- What contexts determine whether the different mechanisms produce their intended outcomes?
- In what circumstances and for whom are VR/AR interventions effective in upskilling health and care providers?

The core research team is a multi-disciplinary group of researchers, from the backgrounds of nursing, primary healthcare, health informatics and implementation. Across this group, expertise relevant to the topic includes that on digital health innovation and evaluation, behaviour change, implementation science and conducting realist reviews. The RAMESES training documents[61] will be referred to, and the review will be reported in accordance with the RAMESES Publication Standards for Realist Synthesis[63] (supplementary Table S1).

Procedures

Realist reviews tend to follow a three-step process: theory elicitation, theory testing, and theory refinement. This process will be followed to describe our procedures. Unlike systematic reviews, which aim to uncover all research relevant to the topic, realist reviews find a comprehensive balance of empirical research and theory[64]. Searches will therefore be iterative and additional rounds of searching may alter the following procedures. Figure 1 highlights the processes that will be conducted in each stage.

For peer review only

1. Theory elicitation

Search strategy

A search will be conducted to identify initial candidate theories. These will not be limited by publication date and are characterised as academic, practitioner and learning and technology adoption theories.

We will identify academic and practitioner theories using free text and MeSH terms when searching Medline, SCOPUS, CINAHL, EMBASE, Education Resource Information Centre, PsycINFO and Web of Science. Snowballing will also help to identify relevant work. Supplementary Table S2 provides the search strategy. An initial search of the databases in January, 2021 located 811 items, of which 200 were deemed potentially eligible, after reviewing their titles and abstracts.

We will focus on the discussion section of items, to identify why AR or VR interventions did or did not achieve their intended outcomes. These often include the author's theories[65]. Existing systematic reviews will first be reviewed.

Relevant practitioner theories may be presented by professional bodies, or within grey literature, including editorials, letters, news articles and commentaries[66]. We will therefore supplement the above search with the additional journals presented in Table 1.

Table 1. Summary of relevant journals related to continued learning in health and care

Professional Body	Journal/s
Association of American Medical Colleges	Academic Medicine; MedEdPORTAL
Association for Medical Education in Europe	Medical Teacher
Foundation for Advancement of International Medical Education and Research	International Journal of Medical Education
Alliance for Continuing Education in the Health Professions; Association for Hospital Medical Education; Society for Academic Continuing Medical Education	Journal of Continuing Education in the Health Professions
German Association for Medical Education	GMS Journal for Medical Education
The Australian & New Zealand Association for Health Professional Educators	Focus on Health Professional Education
Association for the Study of Medical Education	Medical Education
**Journals not associated with a professional body**	

For peer review only

Journal of Nursing Education and Practice
Nurse Education Today
International Journal of Nursing Studies

We will also identify theories related to adult learning from the academic articles. These are expected to include theories related to adult learning for health and care professionals, including the Schon[67] theory on the reflective practitioner, and Slotnick's[68] theory on how physicians learn. Two theories identified by Mukhalalati and Taylor[69] as key to professional learning are directly applicable to AR/VR. These include:

- Experiential learning, whereby knowledge construction and learning are facilitated through interaction with the environment. Kolb[70] proposes a framework for experiential learning that includes concrete experience, reflective observation, abstract conceptualisation, and active experimentation.
- In constructivism, learning occurs through interaction between previous skills/knowledge, those gained through social interaction and social activities, within the learner's environment, physical and social world[71]. Simulation has been identified as a tool that supports constructive learning[72, 73], as constructivists generally believe that people learn best by 'doing,' as this is how they construct their knowledge[74].

Theories may also relate to technology acceptance and adoption. Frameworks include:

- The Diffusion of Innovations theory[75], which explains how and at what rate innovations (e.g. technologies) spread, as determined by different categories of adopters. This can be applied to organisations and individuals.
- The Technology Acceptance Model[76] explains that an individual's perceived usefulness and ease–of-use of a technology influence intention to use and actual use.
- The Unified Theory of Acceptance and Use of Technology[77] determines that four constructs: performance expectancy, effort expectancy; social influence; and, facilitating conditions (e.g. age, gender, experience and voluntariness of use) influence an individual's technology use and acceptance.
- The NASSS framework[78] evaluates reasons for non-adoption, abandonment, and challenges to implementation through six domains (condition, technology, value proposition, adopter system and institutional and societal contexts).
- The Consolidated Framework for Implementation Research[79] considers five domains related to the intervention, outer and inner settings, the individuals involved and implementation process.
- The Normalisation Process Theory[80] focuses on people's actions, rather than their intentions/beliefs. It considers coherence, cognitive participation, collective action and reflexive monitoring as crucial to the implementation process.

<u>Record management</u>

Similar to the methods in Randell et al.[62], records will be saved to an Endnote library, as well as charted on Excel. A timeline sheet on Excel will record search activities, including the databases searched, the date of each search and the number of records found.

For peer review only

Screening

Two researchers (NG and DD) will screen the literature for eligibility, starting by determining the relevance from the title and abstract, and then reading the full-text. The inclusion criteria for the academic and practitioner theories will be:

- Using simulation technologies (any type of immersion will be accepted)
- Health and care workers and individuals post-graduation/registration as learners.
- Any health, care or university-based setting (as these often have simulation labs)
- Includes detail on implementation and/or on what contexts, how and for whom they 'worked.'
- Published in English

The exclusion criteria include simulation technologies that do not use augmentation or VR (e.g. low fidelity web-based e-learning interventions or manikin-only simulators), undergraduate students and published in languages other than English. Work also including undergraduate learners or other simulation technologies will only be included if the data for post-graduate/registered learners and AR/VR can be separated.

A PRISMA flowchart[81] will document the review selection and decision process.

Analysis and synthesis

We will extract relevant information (presented in Box 1) including that pertaining to context, mechanism and outcomes from each article from the academic and practitioner theories. Adult learning and technology adoption theories will be briefly summarised. For consistency, outcomes should broadly be related to the Kirkpatrick et al.[82] components of evaluation: reaction (i.e. satisfaction), learning (i.e. knowledge), behaviour or results (skills). Unintended and other subjective or observed outcomes (e.g. increased confidence or perceived interactivity) will be included too. A second reviewer will code and extract data from a random selection of 10-20% of the articles to ensure consistency in interpretation.

All information will be recorded in an Excel sheet for analysis. If possible, complete CMO configurations will be recorded, however; it is unlikely that all articles will contain complete statements- fragments will therefore be recorded too[62].

Upon completion, we will conduct a narrative synthesis to determine any overlapping CMO configurations. These will then be compared with identified (learning and adoption) theories to further explore the underlying causal mechanisms so as to understand how VR/AR interventions can or should upskill health and care professionals[83]. The resulting CMO configurations will answer (a) what facilitates or constrains the implementation of AR/VR programmes in health and care settings? (b) How, for whom and to what extent did they produce the intended outcomes (reaction/satisfaction, learning/knowledge and behaviour/results)?

The research team will then select a number of CMO configurations to test, focusing on those that are most feasible and likely to apply to future AR or VR interventions.

2. Theory testing

For peer review only

Search strategy

We will search databases to identify empirical and published studies that will enable the CMO configurations to be tested. We will use the same databases and keywords as in step one. Snowballing will help to identify additional literature.

Screening

The articles will be screened by determining their relevance to the programme theory (e.g. AR/VR tools used by health and care workers). A benefit of realist reviews is the focus on the intervention mechanism, enabling the inclusion of literature whereby the intervention has been applied to different settings, people or even similar interventions in the same setting[66, 84]. All study designs will be included. A PRISMA diagram will visualise the study selection process[81].

Analysis and synthesis

Relevant information (presented in Box 1) will be extracted into an excel sheet. We will also assess the quality of each paper using the Mixed Methods Appraisal Tool (MMAT), as this is appropriate for qualitative, quantitative and mixed methods research[85]. The MMAT was developed in 2007[86], and revised in 2011[87]. Unlike earlier versions, the newest 2018 MMAT is not intended to be quantified and instead offers a guide for discussing quality. We will therefore highlight methodological flaws to inform recommendations for future research. Low quality research will not be excluded, as realist methodologists acknowledge that useful information on contextual factors may be present[88]. In alignment with the guidelines for conducting realist reviews, the quality of each study will focus on the evidential fragment (relevant section) that each theory is drawn from[88]. For example, when only quantitative data is used from a mixed-methods study to test the theory, the quality of the qualitative component will not be assessed.

3. Theory refinement

Coded data will be compared to the initial programme theory, and differences will be identified to refine and revise the programme theory. Upon completion of the final theory, a narrative and diagrammatic summary will be presented[89, 90]. We will use the MMAT to assess the extent to which we are confident in each finding. Ultimately, each CMO configuration will be rated as high, moderate, low or very low in confidence. This rating will highlight areas for research and also support decision-makers when deciding whether to implement or develop similar technologies to upskill health and care workers.

Patient and public involvement

Members of the public were not involved in the development of this protocol.

ETHICS, DISSEMINATION AND CONCLUSION

Ethics approval is not required to conduct this realist review. This protocol describes how we will conduct a realist review that constructs, tests and refines an evidence-informed programme theory on what facilitates or constrains the implementation of AR/VR programmes in health and care settings

For peer review only

and how, for whom and to what extent they 'work.' The results may inform and support AR/VR interventions from clinical educators, healthcare providers and software developers. Upskilling through AR/VR learning interventions may ultimately improve quality of care and promote evidence-based practice and continued learning. Findings will be disseminated through conference presentations and peer-reviewed journal publications.

AUTHOR CONTRIBUTIONS

NG conceived and designed the study with support from DD, SNVDV and PW. NG wrote the first draft of the manuscript. All authors revised and approved the final manuscript.

FUNDING

This work is funded by the National Institute for Health Research Applied Research Collaboration Greater Manchester. The views expressed in this publication are those of the authors and not necessarily those of the National Institute for Health Research or the Department of Health and Social Care. Grant number: N/A.

COMPETING INTERESTS

The authors have no competing interests to declare.

PATIENT CONSENT FOR PUBLICATION

Not required.

REFERENCES

1. Ross J. Upskilling could be an ideal treatment for healthcare's employment woes. 2019. https://www.cardiovascularbusiness.com/topics/healthcare-economics/upskilling-could-be-ideal-rx-healthcares-employment-woes.
2. Cavendish C. Cavendish Review: an Independent Enquiry into Healthcare Assistants and Support Workers in the NHS and Social Care Setting. London: Department of Health, 2013.
3. Sarre S, Maben J, Aldus C, et al. The challenges of training, support and assessment of healthcare support workers: A qualitative study of experiences in three English acute hospitals. *International Journal of Nursing Studies* 2018;79:145-53
4. Wright P, Bretthauer K. Strategies for Addressing the Nursing Shortage: Coordinated Decision Making and Workforce Flexibility. *Decision Sciences* 2010;41(2):373-401
5. Fralic M. Upskilling for new-era healthcare: Lessons from high impact readings. *Nurse Leader* 2013;11(4)
6. Rimmer A. Upskill doctors to make the most of new medical technologies, report says. *BMJ* 2018;361(k2861)
7. Almomani E, Sullivan J, Hajjieh M, et al. Simulation-based education programme for upskilling non-critical care nurses for COVID-19 deployment. *BMJ Simulation and Technology Enhanced Learning* 2020
8. NHS. Allied Health Professions. 2020. https://www.england.nhs.uk/ahp/.
9. Brown C, Wickline M, Ecoff L, et al. Nursing practice, knowledge, attitudes and perceived barriers to evidence-based practice at an academic medical centre. *Journal of Advanced Nursing* 2008;65:371–81

For peer review only

10. Dawes M, Summerskill W, Glasziou P, et al. Sicily statement on evidence-based practice. *BMC Medical Education* 2005;5(1):1-7
11. Malik G, McKenna L, Plummer V. Perceived knowledge, skills, attitude and contextual factors affecting evidence-based practice among nurse educators, clinical coaches and nurse specialists. *International Journal of Nursing Practice* 2014;21(S2):46-57
12. Gordon C, Thompson A. Use of personal protective equipment during the COVID-19 pandemic. *British Journal of Nursing* 2020;29(13)
13. Michalec B, Lamb G. COVID-19 and team-based healthcare: The essentiality of theory-driven research. *Journal of Interprofessional Care* 2020;34(5):593-99
14. Portnoy J, Waller M, Elliot T. Telemedicine in the Era of COVID-19. *The Journal of Allergy and Clinical Immunology: In Practice* 2020;8(5):1489-91
15. Wosik J, Fudim M, Cameron B, et al. Telehealth transformation: COVID-19 and the rise of virtual care. *Journal of the American Medical Informatics Association* 2020;27(6):957–62
16. Hatfield T, Withers T, Greaves C. Systematic review of the effect of training interventions on the skills of health professionals in promoting health behaviour, with meta-analysis of subsequent effects on patient health behaviours. *BMC Health Serv Res* 2020;20:593
17. Morris L, Horne M, McEvoy P, et al. Communication training interventions for family and professional carers of people living with dementia: a systematic review of effectiveness, acceptability and conceptual basis. *Aging Ment Health* 2018;22(7):863-80
18. Gerrish K, Clayton J. Promoting evidence-based practice: An organizational approach. *Journal of Nursing Management* 2004;12:114–23
19. Pravikoff D, Tanner A, Pierce S. Readiness of US nurses for evidence-based practice. *The American Journal of Nursing* 2005;105:40–51
20. Thompson C, McCaughan D, Cullum N, et al. Barriers to Evidence-based practice in primary care nursing: Why viewing decision-making as context is helpful. *Journal of Advanced Nursing* 2005;52:432–44
21. Wilberforce M, Abendstern M, Tucker S, et al. Support workers in community mental health teams for older people: roles, boundaries, supervision and training. *J. Adv. Nurs* 2017;73(7):1657-66
22. Curtis K, Brooks S. Digital health technology: Factors affecting implementation in nursing homes. *Nursing Older People* 2020;32(2):14-21
23. Shirey M. Lewin's theory of planned change as a strategic resource. *The Journal of Nursing Administration* 2013;43(92):69–72
24. Keyworth C, Hart J, Armitage C, et al. What maximizes the effectiveness and implementation of technology-based interventions to support healthcare professional practice? A systematic literature review. *BMC Medical Informatics and Decision Making* 2018;18(1):93
25. Austin M, Claassen J. Impact of organizational change on organizational culture. *Journal of Evidence-Informed Social Work* 2008;5(1-2):321–59
26. Udon S, Wagner J. Common Change Theories and Application to Different Nursing Situations. In: Wagner J, ed. Leadership and Influencing Change in Nursing. Canada: University of Regina Press, 2018.
27. Bowers B. Managing change by empowering staff. *Nursing Times* 2011;107(32-33):19–21
28. Spagnoletti C, Bui T, Fischer G, et al. Implementation and evaluation of a web-based communication skills learning tool for training internal medicine interns in patient-doctor communication. *Journal of Communication in Healthcare* 2009;2(2):159-72
29. Yukl G. *Leading in organizations*. 8 ed. Upper Saddle River, NJ: Pearson, 2013.
30. Pottle J. Virtual reality and the transformation of medical education. *Future Healthc J* 2019
31. Seropian M, Brown K, Gavilanes J, et al. Simulation: not just a manikin. *J Nurs Educ* 2004;43(4):164-9
32. Al-Elq A. Simulation-based medical teaching and learning. *Journal of Family & Community Medicine* 2010;17(1):35–40
33. Alamrani M, Alammar K, Alqahtani S, et al. Comparing the Effects of Simulation-Based and Traditional Teaching Methods on the Critical Thinking Abilities and Self-Confidence of Nursing Students. *J Nurs Res* 2018;26(3):152-57

For peer review only

34. Blanié A, Amorim M, Benhamou D. Comparative value of a simulation by gaming and a traditional teaching method to improve clinical reasoning skills necessary to detect patient deterioration: A randomized study in nursing students. *BMC Medical Education* 2020;20(53)

35. William A, Vidal V, John P. Instruction Versus Virtual Reality Simulation: A Comparative Study of Phlebotomy Training among Nursing Students in Kuwait. *Journal of Education and Practice* 2016;7:18-25

36. Maddry J, Varney S, Sessions D, et al. A comparison of simulation-based education versus lecture-based instruction for toxicology training in emergency medicine residents. *Journal of Medical Toxicology: Official Journal of the American College of Medical Toxicology* 2014;10(4):364–68

37. Ziv A, Ben-David S, Ziv M. Simulation based medical education: an opportunity to learn from errors. *Med Teach* 2005;27(3):193-9

38. Freina L, Ott M. Literature review on immersive virtual reality in education: State of the art and perspectives. The International Scientific Conference eLearning and Software for Education, 2015:133.

39. Rogers S. Virtual reality: The learning aid of the 21st century. Secondary Virtual reality: The learning aid of the 21st century 2019.

40. Global Augmented Reality I.G. Global Augmented Reality (AR) and Virtual Reality (VR) Market in Healthcare Market to Reach $11.14 Billion by 2025. 2019. https://www.prnewswire.com/news-releases/global-augmented-reality-ar-and-virtual-reality-vr-market-in-healthcare-market-to-reach-11-14-billion-by-2025--300831306.html.

41. Balian S, McGovern S, Abella B, et al. Feasibility of an augmented reality cardiopulmonary resuscitation training system for health care providers. *5* 2019;8:e02205

42. Bric J, Lumbard D, Frelich M, et al. Current state of virtual reality simulation in robotic surgery training: A review. *Surg Endosc* 2016;30:2169

43. Fleming M, Olsen D, Stathes H, et al. Virtual Reality Skills Training for Health Care Professionals in Alcohol Screening and Brief Intervention. *The Journal of the American Board of Family Medicine* 2009;22(4):387-98

44. Freeman K, Thompson S, Allely E, et al. A Virtual Reality Patient Simulation System for Teaching Emergency Response Skills to U.S. Navy Medical Providers. *Prehospital and Disaster Medicine* 2001;16(1):3-8

45. Harrington C, Kavanagh D, Quinlan J, et al. Development and evaluation of a trauma decision-making simulator in Oculus virtual reality. *Am J Surg* 2018;215:42-47

46. Scerbo M, Bliss J, Schmidt E, et al. The efficacy of a medical virtual realitysimulator for training phlebotomy. *Hum Factors* 2006;48(1):72–84

47. Makransky G, Terkildsen T, Mayer R. Adding immersive virtual reality to a science lab simulation causes more presence but less learning. *Learn. Instr* 2019;60:225–36

48. Makransky G, Andreasen N, Baceviciute S, et al. Immersive virtual reality increases liking but not learning with a science simulation and generative learning strategies promote learning in immersive virtual reality. *J. Educ. Psych* 2020

49. Vidal V, Ohaeri B, John P, et al. Virtual Reality and the Traditional Method for Phlebotomy Training Among College of Nursing Students in Kuwait: Implications for Nursing Education and Practice. *Journal of Infusion Nursing* 2013;36(5):349-55

50. Bowyer M, Pimentel E, Fellows J, et al. Teaching intravenous cannulation to medical students: comparative analysis of two simulators and two traditional educational approaches. *Studies in Health Technology and Informatics* 2005;111:57–63

51. Aïm F, Lonjon G, Hannouche D, et al. Effectiveness of Virtual Reality Training in Orthopaedic Surgery. *Arthroscopy: The Journal of Arthroscopic & Related Surgery* 2016;32(1):224-32

52. Rourke S. How does virtual reality simulation compare to simulated practice in the acquisition of clinical psychomotor skills for pre-registration student nurses? A systematic review. *International Journal of Nursing Studies* 2020;102:103466

53. FundamentalVR. 2020. https://www.fundamentalvr.com.

54. SentiAR. Adding a New Dimension to Clinical Practice. 2020. https://sentiar.com.

55. Augmedics. xVision the future of surgery is within sight. n.d. https://augmedics.com.

For peer review only

56. GLANTA. SureWash Hand Hygiene App. 2020. https://surewash.com/surewash-app-practice-hand-hygiene/.
57. Singh R, Javaid M, Kataria R, et al. Significant applications of virtual reality for COVID-19 pandemic. *Diabetes & metabolic syndrome* 2020;14(4):661–64
58. Horlick K. Virtual reality helps simulation team tackle Covid-19. 2020. https://www.bartshealth.nhs.uk/news/virtual-reality-helps-simulation-team-tackle-covid19--8664.
59. Pawson R, Tilley N. *Realistic Evaluation*. London: SAGE Publications Ltd, 1997.
60. Jagosh J, Macaulay A, Pluye P, et al. Uncovering the benefits of participatory research: implications of a realist review for health research and practice. *Milbank Q* 2012;90:1–41
61. Wong G, Westhorp G, Pawson R, et al. *Realist synthesis. RAMESES training materials*. London: The RAMESES Project, 2013.
62. Randell R, Wright J, Alvarado N, et al. What supports and constrains the implementation of multifactorial falls risk assessment and tailored multifactorial falls prevention interventions in acute hospitals? Protocol for a realist review
63. Wong G, Greenhalgh T, Westhorp G, et al. RAMESES publication standards: realist syntheses. *BMC Med* 2013;11:21
64. Wiese A, Kilty C, Bergin C, et al. Protocol for a realist review of workplace learning in postgraduate medical education and training. *Systematic Reviews* 2017;6(10)
65. Randell R, Honey S, Alvarado N, et al. Embedding robotic surgery into routine practice and impacts on communication and decision making: a review of the experience of surgical teams. *Cognition, Technology & Work* 2016;18(2):423–37
66. Pawson R, Greenhalgh T, Harvey G, et al. Realist review – a new method of systematic review designed for complex policy interventions. *J Health Serv Res Policy* 2005;10(suppl 1):21-34
67. Schon D. *Educating the reflective practitioner: Toward a new design for teaching and learning in the professions*. San Francisco: Jossey-Bass, 1987.
68. Slotnick H. How doctors learn: The role of clinical problems across the Medical School-to-Practice Continuum. *Academic Medicine* 1996:28-34
69. Mukhalalati B, Taylor A. Adult Learning Theories in Context: A Quick Guide for Healthcare Professional Educators. *Journal of Medical Education and Curricular Development* 2019;6
70. Kolb D. *Experiential Learning as the Science of Learning and Development*. Englewood Cliffs, NJ: Prentice Hall, 1984.
71. Kang L, Brian S, Ricca B. Constructivism in pharmacy school. *Curr Pharm Teach Learn* 2010;2(126–130)
72. Greening T. Building the constructivist toolbox: An exploration of cognitive technologies. *Educational Technology* 1998;38(2):28-35
73. Jonassen D, Hernandez-Serrano J, Choi I. Integrating constructivism and learning technologies. In: Spector M, Anderson TM, eds. Integrated and holistic perspectives on learning, instruction and technology: Understanding complexity Netherlands: Kluwer Academic, 2000:103-27.
74. Chen C. Theoretical Bases for Using Virtual Reality in Education. Themes in Science and Technology Education. Klidarithmos Computer Books, 2009.
75. Rogers E. *Diffusion of innovations*. 4 ed. New York: Free Press, 1995.
76. Davis F. Perceived usefulness, perceived ease of use, and user acceptance of information technology. *Management Information Systems Quarterly* 1989;13(3):319-40
77. Venkatesh V, Morris M, Davis G, et al. User acceptance of information technology: Toward a unified view. *Management Information Systems Quarterly* 2003;27(3):425-78
78. Greenhalgh T, Wherton J, Papoutsi C, et al. Beyond Adoption: A New Framework for Theorizing and Evaluating Nonadoption, Abandonment, and Challenges to the Scale-Up, Spread, and Sustainability of Health and Care Technologies. *JMIR* 2017;19(11):e367
79. Damschroder L, Aron D, Keith R, et al. Fostering implementation of health services research findings into practice: a consolidated framework for advancing implementation science. *Implementation Science* 2009;4:50
80. May C, Cummings A, Girling M, et al. Using Normalization Process Theory in feasibility studies and process evaluations of complex healthcare interventions: a systematic review. *Implementation Science* 2018;13(80)

For peer review only

81. Moher D, Liberati A, Tetzlaff J, et al. Preferred reporting items for systematic reviews and meta-analyses: The PRISMA statement. *PLOS Medicine* 2009;6(7):e1000097
82. Kirkpatrick D. *The Four Levels of Evaluation: Measurement and Evaluation*. Alexandria: American Society for Training and Development, 2007.
83. Kislov R, Pope C, Martin G, et al. Harnessing the power of theorising in implementation science. *Implementation Science* 2019;14(1):103
84. Wong G. Data gathering in realist reviews: Looking for needles in haystacks. In: Emmel N, Greenhalgh J, Manzano A, Monaghan M, Dalkin S, eds. Doing realist research. London: Sage, 2018:131-45.
85. Hong Q, FÀBregues S, Bartlett G, et al. The Mixed Methods Appraisal Tool (MMAT) version 2018 for information professionals and researchers. *Education for Information* 2018;34(4):285-91
86. Pluye P, Gagnon M, Griffiths F, et al. A scoring system for appraising mixed methods research, and concomitantly appraising qualitative, quantitative and mixed methods primary studies in Mixed Studies Reviews. *International Journal of Nursing Studies* 2009;46(4):529–46
87. Pace R, Pluye P, Bartlett G, et al. Testing the reliability and efficiency of the pilot Mixed Methods Appraisal Tool (MMAT) for systematic mixed studies review. *International Journal of Nursing Studies* 2012;49(1):47-53
88. Pawson R. Digging for Nuggets: How 'Bad' Research Can Yield 'Good' Evidence. *International Journal of Social Research Methodology* 2006;9(2):127-42
89. Davidoff F, Dixon-Woods M, Leviton L, et al. Demystifying theory and its use in improvement. *BMJ Quality & Safety* 2015;24:228-38
90. Funnell S, Rogers P. *Purposeful program theory: effective use of theories of change and logic models*. San Francisco: John Wiley & Sons, 2011.

FIGURE LEGENDS

Figure 1. Summary of the three steps and process that will be conducted. CMO stands for Context Mechanism and Outcome.

Box 1. Content to be extracted from included sources and recorded in the coding sheet

For peer review only

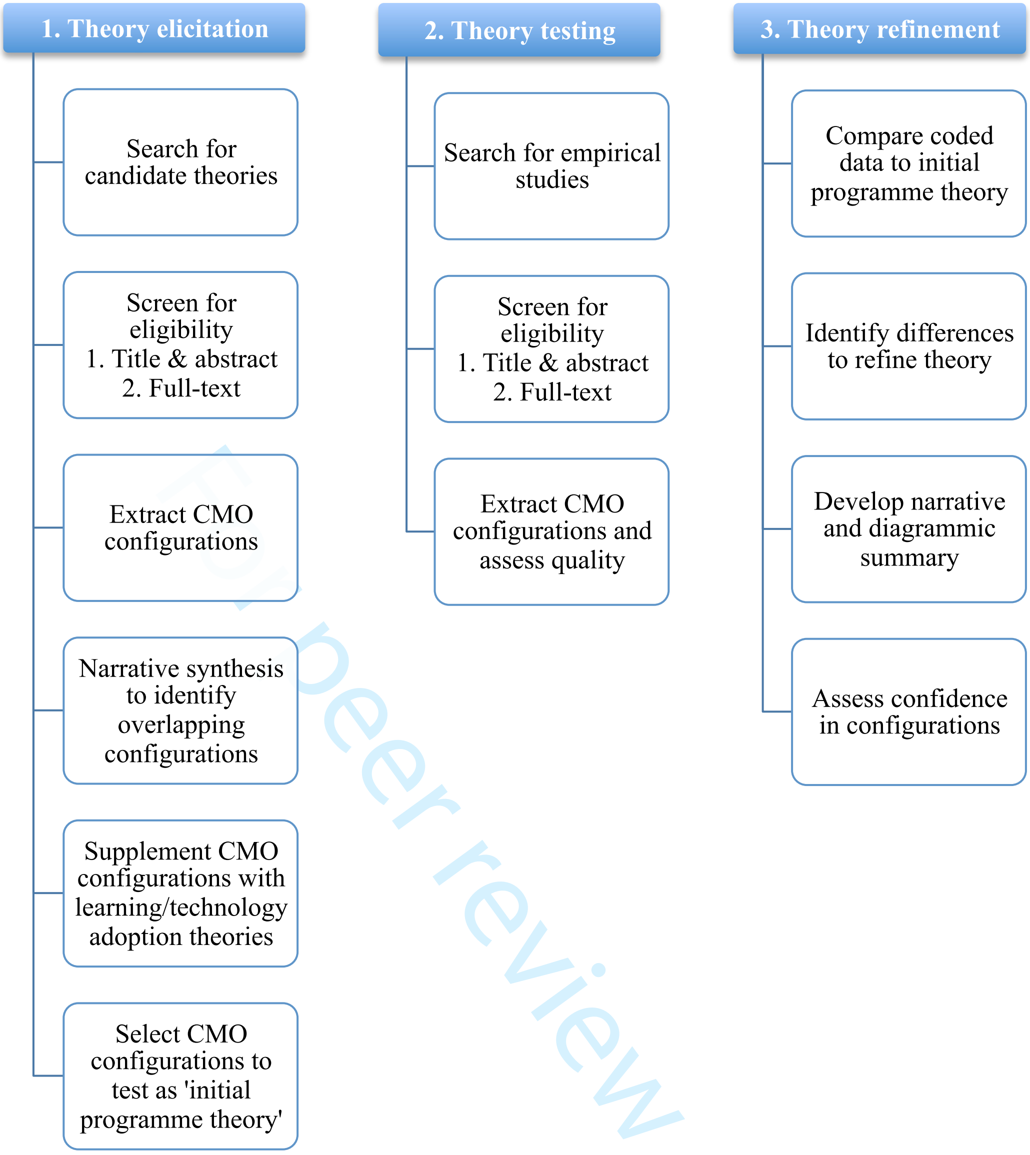

For peer review only

- Author; date
- Title
- Type of publication
- Research design, theoretical orientation (if applicable) and methods
- AR/VR technology description
- Study objective (focus)
- Setting; country
- Sample (type, size, age, gender)
- Context
- Mechanism
- Outcome (intended, unintended and/or subjective)
- Implementation (strategy, adoption and/or uptake)
- Learning or technology adoption theories mentioned

For peer review only

Supplementary file

Table S1. Completed checklist of the RAMESES Publication Standards for realist reviews[1]

**RAMESES* publication standards: realist syntheses<br>* Realist And MEta-narrative Evidence Syntheses: Evolving Standards**				
**No.**	**Section / Topic**	**Checklist item**	**Reported Yes**	**N/A**
**Title**				
**1.**		In the title, identify the document as a realist synthesis or review.	X	
**Abstract**				
**2.**		While acknowledging publication requirements and house style, abstracts should ideally contain brief details of: the study's background, review question or objectives; search strategy; methods of selection, appraisal, analysis and synthesis of sources; main results; and implications for practice	X	
**Introduction**				
**3.**	Rationale for review	Explain why the review is needed and what it is likely to contribute to existing understanding of the topic area.	X	
**4.**	Objectives and focus of review	State the objective(s) of the review and/or the review question(s). Define and provide a rationale for the focus of the review.	X	
**Methods**				
**5.**	Changes in the review process	Any changes made to the review process that was initially planned should be briefly described and justified.		X
**6.**	Rationale for using realist synthesis	Explain why realist synthesis was considered the most appropriate method to use.	X	
**7.**	Scoping the literature	Describe and justify the initial process of exploratory scoping of the literature.	X	
**8.**	Searching processes	While considering specific requirements of the journal or other publication outlet, state and provide a rationale for how the iterative searching was done. Provide details on all the sources accessed for information in the review. Where searching in electronic databases has taken place, the details should include, for example, name of database, search terms, dates of coverage and date last searched. If individuals familiar with the relevant literature and/or topic area were contacted, indicate how they were identified and selected.	X	
**9.**	Selection and appraisal of documents	Explain how judgements were made about including and excluding data from documents, and justify these.	X	
**10.**	Data extraction	Describe and explain which data or information were extracted from the included documents and justify this	X	

		selection.		
**11.**	Analysis and synthesis processes	Describe the analysis and synthesis processes in detail. This section should include information on the constructs analysed and describe the analytic process.	X	
**Results**				
**12.**	Document flow diagram	Provide details on the number of documents assessed for eligibility and included in the review with reasons for exclusion at each stage as well as an indication of their source of origin (for example, from searching databases, reference lists and so on). You may consider using the example templates (which are likely to need modification to suit the data) that are provided.		X
**13.**	Document characteristic s	Provide information on the characteristics of the documents included in the review.		X
**14.**	Main findings	Present the key findings with a specific focus on theory building and testing.		X
**Discussion**				
**15.**	Summary of findings	Summarise the main findings, taking into account the review's objective(s), research question(s), focus and intended audience(s).		X
**16.**	Strengths, limitations and future research directions	Discuss both the strengths of the review and its limitations. These should include (but need not be restricted to) (a) consideration of all the steps in the review process and (b) comment on the overall strength of evidence supporting the explanatory insights which emerged.<br>The limitations identified may point to areas where further work is needed.		X
**17.**	Comparison with existing literature	Where applicable, compare and contrast the review's findings with the existing literature (for example, other reviews) on the same topic.		X
**18.**	Conclusion and recommendat ions	List the main implications of the findings and place these in the context of other relevant literature. If appropriate, offer recommendations for policy and practice.		X
**19.**	Funding	Provide details of funding source (if any) for the review, the role played by the funder (if any) and any conflicts of interests of the reviewers.	X	

[1]Wong, G., Greenhalgh, T., Westhorp, G., et al. RAMESES publication standards: realist syntheses. *BMC Medicine* 2013;11(1):21.

For peer review only

Table S2. Initial search strategy

**Databases**	
	Medline<br>SCOPUS<br>CINAHL<br>EMBASE<br>Education Resource Information Centre<br>PsycINFO<br>Web of Science
**Keywords**	
Technology/Intervention	• augmented reality<br>• virtual reality
Population/sample	• health*<br>healthcare; health; health worker; health staff; health provider<br>• care*<br>care; carer; caregiving; caregiver; caring<br>• nurs*<br>nurse, nursing, nurses<br>• doctor<br>• surgeon
Focus	• training<br>• upskilling<br>• skill<br>• education<br>• evaluation<br>• implementation<br>• feasibility<br>• effectiveness
**Example with Boolean operators**	
	(TITLE-ABS-KEY (augmented AND reality OR virtual AND reality) AND TITLE-ABS-KEY (health* OR care* OR nurs* OR doctor OR surgeon) AND TITLE-ABS-KEY (training OR upskilling OR skill OR education) AND TITLE-ABS-KEY (evaluation OR implementation OR feasibility OR effectiveness))

For peer review only

Table S1. Completed checklist of the RAMESES Publication Standards for realist reviews[1]

**RAMESES* publication standards: realist syntheses<br>* Realist And MEta-narrative Evidence Syntheses: Evolving Standards**				
**No.**	**Section / Topic**	**Checklist item**	**Reported<br>Yes**	**N/A**
**Title**				
**1.**		In the title, identify the document as a realist synthesis or review.	X	
**Abstract**				
**2.**		While acknowledging publication requirements and house style, abstracts should ideally contain brief details of: the study's background, review question or objectives; search strategy; methods of selection, appraisal, analysis and synthesis of sources; main results; and implications for practice	X	
**Introduction**				
**3.**	Rationale for review	Explain why the review is needed and what it is likely to contribute to existing understanding of the topic area.	X	
**4.**	Objectives and focus of review	State the objective(s) of the review and/or the review question(s). Define and provide a rationale for the focus of the review.	X	
**Methods**				
**5.**	Changes in the review process	Any changes made to the review process that was initially planned should be briefly described and justified.		X
**6.**	Rationale for using realist synthesis	Explain why realist synthesis was considered the most appropriate method to use.	X	
**7.**	Scoping the literature	Describe and justify the initial process of exploratory scoping of the literature.	X	
**8.**	Searching processes	While considering specific requirements of the journal or other publication outlet, state and provide a rationale for how the iterative searching was done. Provide details on all the sources accessed for information in the review. Where searching in electronic databases has taken place, the details should include, for example, name of database, search terms, dates of coverage and date last searched. If individuals familiar with the relevant literature and/or topic area were contacted, indicate how they were identified and selected.	X	
**9.**	Selection and appraisal of documents	Explain how judgements were made about including and excluding data from documents, and justify these.	X	
**10.**	Data extraction	Describe and explain which data or information were extracted from the included documents and justify this selection.	X	

For peer review only - http://bmjopen.bmj.com/site/about/guidelines.xhtml

**11.**	Analysis and synthesis processes	Describe the analysis and synthesis processes in detail. This section should include information on the constructs analysed and describe the analytic process.	X	
**Results**				
**12.**	Document flow diagram	Provide details on the number of documents assessed for eligibility and included in the review with reasons for exclusion at each stage as well as an indication of their source of origin (for example, from searching databases, reference lists and so on). You may consider using the example templates (which are likely to need modification to suit the data) that are provided.		X
**13.**	Document characteristic s	Provide information on the characteristics of the documents included in the review.		X
**14.**	Main findings	Present the key findings with a specific focus on theory building and testing.		X
**Discussion**				
**15.**	Summary of findings	Summarise the main findings, taking into account the review's objective(s), research question(s), focus and intended audience(s).		X
**16.**	Strengths, limitations and future research directions	Discuss both the strengths of the review and its limitations. These should include (but need not be restricted to) (a) consideration of all the steps in the review process and (b) comment on the overall strength of evidence supporting the explanatory insights which emerged.<br>The limitations identified may point to areas where further work is needed.		X
**17.**	Comparison with existing literature	Where applicable, compare and contrast the review's findings with the existing literature (for example, other reviews) on the same topic.		X
**18.**	Conclusion and recommendat ions	List the main implications of the findings and place these in the context of other relevant literature. If appropriate, offer recommendations for policy and practice.		X
**19.**	Funding	Provide details of funding source (if any) for the review, the role played by the funder (if any) and any conflicts of interests of the reviewers.	X	

[1]Wong, G., Greenhalgh, T., Westhorp, G., et al. RAMESES publication standards: realist syntheses. *BMC Medicine* 2013;11(1):21.

For peer review only

BMJ Open

Upskilling health and care workers with augmented and virtual reality: Protocol for a realist review to develop an evidence-informed programme theory

Journal:	*BMJ Open*
Manuscript ID	bmjopen-2021-050033.R1
Article Type:	Protocol
Date Submitted by the Author:	24-May-2021
Complete List of Authors:	Gasteiger, Norina; The University of Manchester Faculty of Biology Medicine and Health, Division of Nursing, Midwifery & Social Work; The University of Manchester Faculty of Biology Medicine and Health, Centre for Health Informatics, Division of Informatics, Imaging and Data Sciences<br>van der Veer, Sabine N.; The University of Manchester Faculty of Biology Medicine and Health, Centre for Health Informatics, Division of Informatics, Imaging and Data Sciences; The University of Manchester, Manchester Academic Health Science Centre<br>Wilson, Paul; The University of Manchester Faculty of Biology Medicine and Health, Division of Population Health, Health Services Research & Primary Care<br>Dowding, Dawn; The University of Manchester Faculty of Biology Medicine and Health, Division of Nursing, Midwifery & Social Work
**Primary Subject Heading**:	Medical education and training
Secondary Subject Heading:	Health informatics
Keywords:	MEDICAL EDUCATION & TRAINING, EDUCATION & TRAINING (see Medical Education & Training), Information technology < BIOTECHNOLOGY & BIOINFORMATICS, Health informatics < BIOTECHNOLOGY & BIOINFORMATICS

SCHOLARONE™
Manuscripts

I, the Submitting Author has the right to grant and does grant on behalf of all authors of the Work (as defined in the below author licence), an exclusive licence and/or a non-exclusive licence for contributions from authors who are: i) UK Crown employees; ii) where BMJ has agreed a CC-BY licence shall apply, and/or iii) in accordance with the terms applicable for US Federal Government officers or employees acting as part of their official duties; on a worldwide, perpetual, irrevocable, royalty-free basis to BMJ Publishing Group Ltd ("BMJ") its licensees and where the relevant Journal is co-owned by BMJ to the co-owners of the Journal, to publish the Work in this journal and any other BMJ products and to exploit all rights, as set out in our licence.

The Submitting Author accepts and understands that any supply made under these terms is made by BMJ to the Submitting Author unless you are acting as an employee on behalf of your employer or a postgraduate student of an affiliated institution which is paying any applicable article publishing charge ("APC") for Open Access articles. Where the Submitting Author wishes to make the Work available on an Open Access basis (and intends to pay the relevant APC), the terms of reuse of such Open Access shall be governed by a Creative Commons licence – details of these licences and which Creative Commons licence will apply to this Work are set out in our licence referred to above.

Other than as permitted in any relevant BMJ Author's Self Archiving Policies, I confirm this Work has not been accepted for publication elsewhere, is not being considered for publication elsewhere and does not duplicate material already published. I confirm all authors consent to publication of this Work and authorise the granting of this licence.

For peer review only

Upskilling health and care workers with augmented and virtual reality: Protocol for a realist review to develop an evidence-informed programme theory

Norina Gasteiger[1,2,3,*], Sabine N van der Veer[2,4], Paul Wilson[3] and Dawn Dowding[1]

1. Division of Nursing, Midwifery & Social Work, University of Manchester, Manchester, UK

2. Centre for Health Informatics, Division of Informatics, Imaging and Data Sciences, University of Manchester, Manchester, UK

3. Division of Population Health, Health Services Research & Primary Care, University of Manchester, Manchester, UK

4. Manchester Academic Health Science Centre, University of Manchester, Manchester, UK

***Corresponding author:**
Norina Gasteiger
Location: Jean McFarlane Building, Oxford Road, Manchester, M13 9PL, United Kingdom
Email: norina.gasteiger@postgrad.manchester.ac.uk

ORCiD:
N. Gasteiger: 0000-0001-7801-7417
S.N van der Veer: 0000-0003-0929-436X
P. Wilson: 0000-0002-2657-5780
D. Dowding: 0000-0001-5672-8605

For peer review only

ABSTRACT

Introduction

Augmented reality (AR) and virtual reality (VR) are increasingly used to upskill health and care providers, including in surgical, nursing and acute care settings. Many studies have used AR/VR to deliver training, providing mixed evidence on their effectiveness and limited evidence regarding contextual factors that influence effectiveness and implementation. This review will develop, test and refine an evidence-informed programme theory on what facilitates or constrains the implementation of AR or VR programmes in health and care settings and understand how, for whom and to what extent they 'work.'

Method and analysis

This realist review adheres to the RAMESES standards and will be conducted in three steps: theory elicitation, theory testing, and theory refinement. First, a search will identify practitioner, academic and learning and technology adoption theories from databases (Medline, SCOPUS, CINAHL, EMBASE, Education Resource Information Centre, PsycINFO and Web of Science), practitioner journals, snowballing and grey literature. Information regarding context, mechanism and outcome will be extracted. A narrative synthesis will determine overlapping configurations and form an initial theory. Second, the theory will be tested using empirical evidence located from the above databases and identified from the first search. Quality will be assessed using the Mixed Methods Appraisal Tool (MMAT), and relevant information will be extracted into a coding sheet. Third, the extracted information will be compared to the initial programme theory, with differences helping to make refinements. Findings will be presented as a narrative summary, and the MMAT will determine our confidence in each configuration.

Ethics and dissemination

Ethics approval is not required. This review will develop an evidence-informed programme theory. The results will inform and support AR/VR interventions from clinical educators, healthcare providers and software developers. Upskilling through AR/VR learning interventions may improve quality of care and promote evidence-based practice and continued learning. Findings will be disseminated through conference presentations and peer-reviewed journal articles.

KEYWORDS

healthcare; learning; simulation; virtual reality; augmented reality; realist review

STRENGTHS AND LIMITATIONS OF THIS STUDY

- Including quality assessments and identifying our confidence in each CMO configuration will improve applicability of the programme theory.
- The repeated search will help to include recently published and up-to-date literature.
- This review will be conducted systematically, which enhances reproducibility.
- The literature review may be subject to selection bias, because it will only include published, peer-reviewed studies in English.
- The mechanisms extracted will likely be untested and subjective author hypotheses.

WORD COUNT: 4,397

For peer review only

INTRODUCTION

Upskilling in the health and care workforce

Upskilling through continuous learning and development is important in any business, to improve skillsets, advance practice and close gaps in knowledge. Upskilling is the process of learning new skills or refining existing skillsets to enable employees to continue practising with ease[1]. For health support and care workers, upskilling ensures that their work is safe and aligns with best practice guidelines, as they often receive variable and inconsistent training, as non-registered staff[2,3]. Upskilling, in this sense, is therefore essential for providing consistent and high-quality care. Additionally, this promotes workforce flexibility and enables for the delegation of skills, when systems experience a shortage of staff[4]. Within the provision of health and care, upskilling is also crucial when adapting in times of change[5,6] or crisis[7] and to align with up-to-date best practice.

Health and care providers may range from registered clinicians such as surgeons, general practitioners and doctors, nurses and midwives, to allied health and non-registered staff who provide care. Allied health staff may include paramedics, dieticians, podiatrists and radiographers[8], while carers also include those working for care-based organisations such as in care homes or homecare agencies. Regardless of the role, all staff that provide health and care services must act in accordance with policies/guidelines and optimally engage in up-to-date evidence-based best practice.

Evidence-based practice is widely recognised as the gold standard when providing effective and safe healthcare[9]. This requires professionals to update and upskill themselves on current evidence and to alter their practice to align with this, as well as with their patient's preferences[10]. Current evidence is usually retrieved from peer-reviewed journal articles; however, due to time constraints and workload demand, many health and care staff rely on organisational policies and protocols as formal sources of knowledge[11]. As the evidence base grows, old habits must be adapted and upskilling is required to align with the newest best practice.

Upskilling is also essential when adapting in times of change or crisis. For example, the emergence of medical and healthcare technologies requires staff to upskill, including improving their digital literacy skills[5,6]. Additionally, the novel coronavirus disease-19 (COVID-19) pandemic caused significant changes to health and care systems. Changes included staff deployment to wards (e.g. COVID-19 wards) outside of their normal experience and of retired and newly qualified staff, remote provision of healthcare using telehealth (phones, video, patient portals), distancing/minimal contact care, stringent use of personal protective equipment and strengthened inter-professional collaboration[12-15]. These challenges required prompt upskilling, especially in using technologies and in infection, prevention and control behaviours to minimise the spread of COVID-19.

Upskilling training programmes

Upskilling training programmes traditionally consist of e-learning, textbooks, workshops, seminars, shadowing/observation and reading peer-reviewed journal articles. Hatfield et al.[16] systematically reviewed 12 studies that delivered behaviour change training interventions to healthcare professionals. All used educational elements (e.g. presentations and workshops) and most were delivered in-person. Morris et al.[17] reviewed training interventions aimed at carers. Both reviews concluded that interventions that use both educational and practical elements (e.g. practising skills or discussion) are

For peer review only

most effective [16,17]. This indicates that education-only interventions may not be effective in upskilling health and care staff.

Time, organisational structure, difficult to access resources and a reliance on experiential knowledge also constrain providers from upskilling[3,11]. Health and care staff have widely reported a preference for learning through 'doing' (such as interacting with or observing colleagues), rather than from journal articles or textbooks[18-20]. Additionally, although support and care workers provide clinical, care-based and clerical patient care, their value is often not reflected in their allocated training budgets and available programmes[3]. As a result, many feel insufficiently prepared[3]. However, clinical, health support and care staff indicate a willingness to upskill, receive further training and to participate in interventions that will improve their practice[11,21]. Further, some managers and nurses in England-based nursing homes have expressed enthusiasm toward implementing innovative digital health technologies that may improve residents' quality of care[22].

Digital technologies for upskilling

Effective interventions that are short, accessible, interactive, memorable and low cost are needed, to overcome training barriers. For workplaces with staff shortages, training also needs to be flexible and provided on a drop-by basis[3]. Brief interventions delivered via digital technology may be appropriate, as they can be made available online and accessed 24/7. They can also be more engaging and memorable, by including interactive activities (e.g. games, quizzes, simulations and immediate performance feedback). However, there is limited literature on implementation strategies for digital interventions that upskill health and care workers. Theories of change can be applied to knowledge of existing barriers and facilitators to using digital health programmes for healthcare workers. Lewin describes behaviour as "a dynamic balance of forces working in opposing directions" [23]. Lewin theorises that driving forces (i.e. facilitators) and restraining forces (i.e. barriers) counter one another, but can result in change if one overrides the other. This means that barriers and facilitators directly impact the implementation success and effectiveness of digital training programmes for health and care staff.

Literature on digital health technologies has highlighted various driving and restraining forces that impact both implementation and the effectiveness of programmes. Keyworth et al.[24] conducted a review of 69 studies to determine what maximises the effectiveness and implementation of technology-based interventions that support healthcare professional practice. They concluded that successful technologies employ behaviour change theories and specific instruction on how to perform behaviours. They also provide professionals with knowledge and person-specific information to assist with practice (e.g. patient management). Driving forces for implementation include integration into clinical workload, alignment with organisational strategies and senior peer endorsement. Restraining forces include organisational challenges, as well as the design, content and technical issues of the interventions.

Literature also highlights key strategies for implementation, focussing on provider adoption and acceptance. Recommendations for facilitating change include linking new practice with old practice to build familiarity[25,26], identifying people who are willing to facilitate and promote the new practice[26,27] and to clearly communicate to staff as to how the new practice will benefit them and their patients[26,28,29]. Spagnoletti et al.[28] provide specific examples, highlighting that short sessions, role-modelling content (e.g. video clips of the behaviour) and modules that refresh

For peer review only

understanding of familiar curriculum were important in their implementation of an online training programme for interns.

Simulation technologies for upskilling

The implementation of simulation technologies may be a novel and engaging approach to upskilling health and care workers. The term health and care workers captures the breadth of professionals working in health and social care, including medical staff, general practitioners, nurses, carers and community workers. Simulation in this context refers to the replication of real-life interactions or scenarios, whereby learners receive immediate feedback/de-briefing[30]. Various levels of simulation exist, depending on 'fidelity' (reality). According to Seropian et al.[31], these can be categorised as high, medium and low fidelity and use tools such as human-like body parts, haptic feedback, computer programmes (e.g. serious games) or virtual reality (VR) headsets to facilitate experimental learning. Low fidelity simulation may include a simple body part, such as a doll-like arm to practice intravenous insertion skills[32]. In contrast, high fidelity simulation tools include real-life responses driven by computers[32]. These are more expensive and may include the METI Human Patient Simulator, which looks and acts like a human (e.g. blinks, has a pulse and speaks) and accurately mirrors responses to clinical procedures, such as intubation and catheterisation. However, it is important to note that simulators mimic, rather than replicate reality[32].

Simulation technology has been found to be as effective as traditional teaching methods for educating health and care staff and students[33-35]. However, when compared to traditional methods, students report better retention of knowledge[36] and higher satisfaction and motivation when using simulation technologies such as games[34]. Experimental learning by simulation also allows for learners to repeatedly practice skills and make and learn from their mistakes without harming a patient, distressing them or facing other negative consequences[32,37]. Computer-driven simulation technologies such as games, augmentation and VR also enable independent learning, often without the need for an instructor to immediately provide feedback or de-brief learners. De-briefing can then occur at a later date, such as to determine trainee performance and learning progress.

In VR, users wear a headset to become immersed in a digital environment. Headsets range from the low-cost Samsung Gear VR or Google Cardboard to high-end gaming equipment such as Oculus Touch. The extent of immersion also differs, ranging from non-immersion (e.g. using computer-based VR), semi-immersion and fully immersive simulations (e.g. those with haptic feedback). The perception of being immersed within a non-physical world is created through various stimuli, including images and sound[38], which enable users to learn from experience. In interactive medical VR, users can engage in virtual worlds, including with patients, colleagues and react to specific scenarios[30]. In contrast, within augmented reality (AR) real-world environments are complemented with interactive computer-generated imagery and information.

Unlike traditional simulators, the main benefit of VR is transporting the learner into an immersive environment. VR and AR interventions are also cost-effective as they can be used autonomously, independently, and repeatedly, compared to traditional simulation technologies. In fact, they have been deemed as the learning tool of the 21st century[39] and their popularity is expected to continually increase. Current projections for the AR/VR head-mounted display market include a worth of USD 25 billion by 2022, with an annual growth rate of 39.5%[40]. This highlights that now is the ideal time to research implementation of AR/VR, due to an inevitable growth in use and further reduction in costs.

For peer review only

These technologies have transformed clinical training and have been used to support health care workers in decision-making and teaching emergency response, resuscitation, robotic surgery and alcohol screening skills[41-45]. However, their effectiveness is contested within the literature, with some research stating that VR is not as effective as other training tools, including for phlebotomy training[46]. Other literature highlights that VR is useful for 'presence', but does not improve learning outcomes[47,48]. It is hypothesised that VR increases cognitive load and therefore compromises cognitive resources from the learning experience[47]. Conversely, some research has found VR to be more effective than other educational techniques[49,50], with systematic reviews concluding that VR training is effective in improving technical skills for arthroscopic surgery[51] and knowledge and skill performance when learning clinical psychomotor skills[52]. Evidently, research is needed to explore to what extent and for whom VR interventions are effective.

Despite their contested effectiveness, VR and AR technologies have now been commercialised and implemented to upskill and support health providers. FundamentalVR[53], for example, provides flight simulator-like training for surgeons with the use of haptic elements for tactile feedback. In the SentiAR[54] tool, holographic visualisations are provided for each patients anatomy and float alongside or above the patient during procedures (e.g. treating cardiac arrhythmias). Other tools include the AR xVision[55] three-dimensional anatomical images that enable clinical providers to see a patient's skin and tissue (akin to x-ray vision) and the AR SureWash[56] mobile app, which provides personalised feedback for hand hygiene technique[57]. VR technologies were also implemented during the COVID-19 pandemic when face-to-face teaching was not possible[58]. For example, St Bartholomew's Hospital used VR to train their nurses and doctors on 50 clinical procedures[59]. Their OMS VR system provided performance feedback, tracked improvement and facilitated group learning.

Gap in research and aim

Despite the emergence and potential efficacy of simulation technologies, the effectiveness of these technologies as an educational intervention remains debated. This includes how good they are at enabling upskilling compared to other strategies, and how they can be implemented into a practice setting, to enable upskilling. Additionally, as evident in the mixed findings on the effectiveness of AR and VR interventions in upskilling staff, programme interventions, including digital ones, do not work for everyone equally[60]. A gap in research remains on the factors that influence when an AR or VR intervention works, to what extent, for whom and in which context. Moreover, research is needed on the causal mechanisms that influence the outcomes of AR/VR interventions and their implementation. This is essential in ensuring that future digital interventions are designed and appropriately targeted at health and care workers, for both maximum efficiency and sustained effects. The aim of this review is to develop, test and refine an evidence-informed programme theory on what facilitates or constrains the implementation of AR or VR programmes in health and care settings and understand how, for whom and to what extent they 'work.'

METHODS AND ANALYSIS

Realist review

This research will take a realist approach because it can produce useful answers to complex questions often left unexplored by experimental research[60]. These questions include: how, when, for whom

For peer review only

and to what extent, does an intervention 'work'? To answer these questions, realist approaches consider the complex interactions between the environment, individuals and the intervention.

Realist evaluation is an emerging theory-driven methodology that seeks to understand CMO configurations, i.e. the context (C), mechanisms (M) and outcomes (O) of interventions. Context refers to the backdrop of conditions that may impact outcomes, such as organisational structure, functional fidelity, environmental settings, culture and norms. These trigger or modify mechanisms (causal forces) that influence outcomes[61]. Examples of mechanisms include the resources offered by interventions or changes in reasoning or behaviour.

Realist reviews seek to understand context, mechanisms and outcomes, by identifying candidate theories and then systematically reviewing literature for underlying social entities, processes or social structures that result in the intended outcome[62]; rather than assuming that the intervention itself produces an outcome. This process is useful for complex interventions, in which outcomes may not necessarily be linear, and instead depend on the context and both intended and unintentional mechanisms[62]. It also allows exploring how an intervention is meant to work compared to how it actually works in practice[63]. Additionally, 'demi-regularities' are identified to acknowledge that outcomes will vary across contexts, but some CMO patterns will remain[61]. This focuses reviewers on the transferable aspects of a programme theory[62]. By definition, candidate theories are individual and specific theories, while a programme theory provides an over-arching explanation of how a specific intervention is expected to 'work,' including how contexts and mechanisms lead to negative and positive outcomes[64].

CMO configurations are then developed as a programme theory, which is tested and refined in real-life settings and with key stakeholders[60]. As with AR/VR technologies, the main benefit of realist evaluation is the ability to bridge theory and practical application in the contexts and with the populations that the intervention targets[60].

A realist review will therefore help to answer the following questions:

- What facilitates or constrains the implementation of AR/VR programmes in health and care settings?
- What are the mechanisms by which VR/AR interventions result in their intended outcomes?
- What contexts determine whether the different mechanisms produce their intended outcomes?
- In what circumstances and for whom are VR/AR interventions effective in upskilling health and care providers?

The core research team is a multi-disciplinary group of researchers, from the backgrounds of nursing, primary healthcare, health informatics and implementation. Across this group, expertise relevant to the topic includes that on digital health innovation and evaluation, behaviour change, implementation science and conducting realist reviews. The RAMESES training documents[62] will be referred to, and the review will be reported in accordance with the RAMESES Publication Standards for Realist Synthesis[65] (supplementary Table S1).

Procedures

Realist reviews tend to follow a three-step process: theory elicitation, theory testing, and theory refinement. This process will be followed to describe our procedures. Unlike systematic reviews,

For peer review only

which aim to uncover all research relevant to the topic, realist reviews find a comprehensive balance of empirical research and theory[66]. Searches will therefore be iterative and additional rounds of searching may alter the following procedures. Figure 1 highlights the processes that will be conducted in each stage.

1. Theory elicitation

Search strategy

A search will be conducted to identify initial candidate theories. These will not be limited by publication date and are characterised as academic, practitioner and learning and technology adoption theories.

We will identify academic and practitioner theories using free text and MeSH terms when searching Medline, SCOPUS, CINAHL, EMBASE, Education Resource Information Centre, PsycINFO and Web of Science. Snowballing will also help to identify relevant work. Supplementary Table S2 provides the search strategy. An initial search of the databases in January, 2021 located 811 items, of which 200 were deemed potentially eligible, after reviewing their titles and abstracts.

We will focus on the discussion section of items, to identify why AR or VR interventions did or did not achieve their intended outcomes. These often include the author's theories[67]. Existing systematic reviews will first be reviewed.

Relevant practitioner theories may be presented by professional bodies, or within grey literature, including editorials, letters, news articles and commentaries[68]. We will therefore supplement the above search with the additional journals presented in Table 1. Literature from other relevant journals such as JMIR and JAMIA will be identified through the database searches, as they are indexed in Medline.

Table 1. Summary of relevant journals related to continued learning in health and care

Professional Body	Journal/s
Association of American Medical Colleges	Academic Medicine; MedEdPORTAL
Association for Medical Education in Europe	Medical Teacher
Foundation for Advancement of International Medical Education and Research	International Journal of Medical Education
Alliance for Continuing Education in the Health Professions; Association for Hospital Medical Education; Society for Academic Continuing Medical Education	Journal of Continuing Education in the Health Professions
German Association for Medical Education	GMS Journal for Medical Education

For peer review only

The Australian & New Zealand Association for Health Professional Educators	Focus on Health Professional Education
Association for the Study of Medical Education	Medical Education
**Journals not associated with a professional body**	
Journal of Nursing Education and Practice	
Nurse Education Today	
International Journal of Nursing Studies	

We will also identify theories related to adult learning from the academic articles. These are expected to include theories related to adult learning for health and care professionals, including the Schon[69] theory on the reflective practitioner, and Slotnick's[70] theory on how physicians learn. Two theories identified by Mukhalalati and Taylor[71] as key to professional learning are directly applicable to AR/VR. These include:

- Experiential learning, whereby knowledge construction and learning are facilitated through interaction with the environment. Kolb[72] proposes a framework for experiential learning that includes concrete experience, reflective observation, abstract conceptualisation, and active experimentation.
- In constructivism, learning occurs through interaction between previous skills/knowledge, those gained through social interaction and social activities, within the learner's environment, physical and social world[73]. Simulation has been identified as a tool that supports constructive learning[74,75], as constructivists generally believe that people learn best by 'doing,' as this is how they construct their knowledge[76].

Theories may also relate to technology acceptance and adoption. Frameworks include:

- The Diffusion of Innovations theory[77], which explains how and at what rate innovations (e.g. technologies) spread, as determined by different categories of adopters. This can be applied to organisations and individuals.
- The Technology Acceptance Model[78] explains that an individual's perceived usefulness and ease–of-use of a technology influence intention to use and actual use.
- The Unified Theory of Acceptance and Use of Technology[79] determines that four constructs: performance expectancy, effort expectancy; social influence; and, facilitating conditions (e.g. age, gender, experience and voluntariness of use) influence an individual's technology use and acceptance.
- The NASSS framework[80] evaluates reasons for non-adoption, abandonment, and challenges to implementation through six domains (condition, technology, value proposition, adopter system and institutional and societal contexts).
- The Consolidated Framework for Implementation Research[81] considers five domains related to the intervention, outer and inner settings, the individuals involved and implementation process.

For peer review only

- The Normalisation Process Theory[82] focuses on people's actions, rather than their intentions/beliefs. It considers coherence, cognitive participation, collective action and reflexive monitoring as crucial to the implementation process.

Record management

Similar to the methods in Randell et al.[63], records will be saved to an Endnote library, as well as charted on Excel. A timeline sheet on Excel will record search activities, including the databases searched, the date of each search and the number of records found.

Screening

Two researchers (NG and DD) will screen the literature for eligibility, starting by determining the relevance from the title and abstract, and then reading the full-text. As in other realist reviews, the first researcher will screen all items and generate a short-list of possible eligible items, while the second independently screens a random sub-set of items (20%) at each screening stage[83]. A raw agreement rate will be calculated to determine interrater reliability, while any disagreements will be resolved through discussion, so that consensus is met. The inclusion criteria for the academic and practitioner theories will be:

- Using simulation technologies (any type of immersion will be accepted)
- Health and care workers and individuals post-graduation/registration as learners.
- Any health, care or university-based setting (as these often have simulation labs)
- Includes detail on implementation and/or on what contexts, how and for whom they 'worked.'
- Published in English

The exclusion criteria include simulation technologies that do not use augmentation or VR (e.g. low fidelity web-based e-learning interventions or manikin-only simulators), undergraduate students and published in languages other than English. Work also including undergraduate learners or other simulation technologies will only be included if the data for post-graduate/registered learners and AR/VR can be separated. Undergraduate students will be excluded as they differ from learners post-registration. Namely, they are learning content for the first time, rather than upskilling their clinical or practical knowledge/experience. For the purpose of this review, VR is defined as a computer-generated simulated environment, while AR refers to the projection of computer-generated imagery onto real-world environments[84,85].

A PRISMA flowchart[86] will document the review selection and decision process.

Analysis and synthesis

We will extract relevant information (presented in Box 1) including that pertaining to context, mechanism and outcomes from each article from the academic and practitioner theories. Adult learning and technology adoption theories will be briefly summarised. For consistency, outcomes should broadly be related to the Kirkpatrick et al.[87] components of evaluation: reaction (i.e. satisfaction), learning (i.e. knowledge), behaviour or results (skills). Unintended and other subjective or observed outcomes (e.g. increased confidence or perceived interactivity) will be included too. A second reviewer will code and extract data from a random selection of 10-20% of the articles to ensure consistency in interpretation.

For peer review only

Box 1. Content to be extracted from included sources and recorded in the coding sheet

- Author; date
- Title
- Type of publication (journal paper, conference paper or book chapter)
- Research design, theoretical orientation (if applicable) and methods
- AR/VR technology description
- Study objective (focus)
- Setting; country
- Sample (type, size, age, gender)
- Context
- Mechanism
- Outcome (intended, unintended and/or subjective)
- Implementation (strategy, adoption and/or uptake)
- Learning or technology adoption theories mentioned (if applicable)

All information will be recorded in an Excel sheet for analysis. If possible, complete CMO configurations will be recorded, however; it is unlikely that all articles will contain complete statements- fragments will therefore be recorded too[63].

Upon completion, we will conduct a narrative synthesis to determine any overlapping CMO configurations. These will then be compared with identified (learning and adoption) theories to further explore the underlying causal mechanisms so as to understand how VR/AR interventions can or should upskill health and care professionals[88]. The resulting CMO configurations will answer (a) what facilitates or constrains the implementation of AR/VR programmes in health and care settings? (b) How, for whom and to what extent did they produce the intended outcomes (reaction/satisfaction, short and long-term learning/knowledge and behaviour/results)?

The research team will then select a number of CMO configurations to test, focusing on those that are most feasible and likely to apply to future AR or VR interventions.

2. Theory testing

<u>Search strategy</u>

We will search databases to identify empirical and published studies that will enable the CMO configurations to be tested. First we will identify the empirical literature from step one. We will then search the same databases as in step one, using the same keywords, but limit the timeframe of the search to only include recently published literature that we will have missed since conducting the first search. Snowballing will help to identify additional literature. This will consist of checking the reference lists of the included literature.

<u>Screening</u>

For peer review only

The articles will be screened by determining their relevance to the programme theory (e.g. AR/VR tools used by health and care workers). A benefit of realist reviews is the focus on the intervention mechanism, enabling the inclusion of literature whereby the intervention has been applied to different settings, people or even similar interventions in the same setting[68,89]. All study designs will be included. A PRISMA diagram will visualise the study selection process[86].

Analysis and synthesis

Relevant information (presented in Box 1) will be extracted into an excel sheet. We will also assess the quality of each paper using the Mixed Methods Appraisal Tool (MMAT), as this is appropriate for qualitative, quantitative and mixed methods research[90]. The MMAT was developed in 2007[91], and revised in 2011[92]. Unlike earlier versions, the newest 2018 MMAT is not intended to be quantified and instead offers a guide for discussing quality. We will therefore highlight methodological flaws to inform recommendations for future research. Low quality research will not be excluded, as realist methodologists acknowledge that useful information on contextual factors may be present[93]. In alignment with the guidelines for conducting realist reviews, the quality of each study will focus on the evidential fragment (relevant section) that each theory is drawn from[93]. For example, when only quantitative data is used from a mixed-methods study to test the theory, the quality of the qualitative component will not be assessed. Cohen's kappa will be calculated, to determine interrater reliability between the two authors conducting the quality assessments.

3. Theory refinement

Coded data will be compared to the initial programme theory, and differences will be identified to refine and revise the programme theory. Upon completion of the final theory, a narrative and diagrammatic summary will be presented[64,94]. We will use the MMAT to assess the extent to which we are confident in each finding. Ultimately, each CMO configuration will be rated as high, moderate, low or very low in confidence. This rating will highlight areas for research and also support decision-makers when deciding whether to implement or develop similar technologies to upskill health and care workers.

Strengths and limitations

Inherent limitations of realist reviews must be acknowledged. Realist reviews have been critiqued to be laborious and time-intensive[95], so the included literature is not always up-to-date when it is published. We will overcome this through a second database search, which will specifically identify recently published work. Programme theories are also only as good as the literature they include, but they do sometimes not acknowledge or assess quality[83]. We are therefore conducting quality assessments of the literature and using this to identify our confidence in each CMO configuration. A fundamental limitation we cannot overcome but must acknowledge is that mechanisms are often untested and subjective author hypotheses[96], which may limit the accuracy of the programme theory.

Patient and public involvement

Members of the public were not involved in the development of this protocol.

ETHICS, DISSEMINATION AND CONCLUSION

Ethics approval is not required to conduct this realist review. This protocol describes how we will conduct a realist review that constructs, tests and refines an evidence-informed programme theory on what facilitates or constrains the implementation of AR/VR programmes in health and care settings and how, for whom and to what extent they 'work.' The results may inform and support AR/VR interventions from clinical educators, healthcare providers and software developers. Upskilling through AR/VR learning interventions may ultimately improve quality of care and promote evidence-based practice and continued learning. Findings will be disseminated through conference presentations and peer-reviewed journal publications. In our future work we will continue to refine our programme theory, by involving stakeholders. This will include interviews, as well as experimental work.

AUTHOR CONTRIBUTIONS

NG conceived and designed the study with support from DD, SNVDV and PW. NG wrote the first draft of the manuscript. All authors revised and approved the final manuscript.

FUNDING

This work is funded by the National Institute for Health Research Applied Research Collaboration Greater Manchester. The views expressed in this publication are those of the authors and not necessarily those of the National Institute for Health Research or the Department of Health and Social Care. Grant number: N/A.

COMPETING INTERESTS

The authors have no competing interests to declare.

PATIENT CONSENT FOR PUBLICATION

Not required.

REFERENCES

1. Ross J. Upskilling could be an ideal treatment for healthcare's employment woes. 2019. https://www.cardiovascularbusiness.com/topics/healthcare-economics/upskilling-could-be-ideal-rx-healthcares-employment-woes.
2. Cavendish C. Cavendish Review: an Independent Enquiry into Healthcare Assistants and Support Workers in the NHS and Social Care Setting. London: Department of Health, 2013.
3. Sarre S, Maben J, Aldus C, et al. The challenges of training, support and assessment of healthcare support workers: A qualitative study of experiences in three English acute hospitals. *International Journal of Nursing Studies* 2018;79:145-53
4. Wright P, Bretthauer K. Strategies for Addressing the Nursing Shortage: Coordinated Decision Making and Workforce Flexibility. *Decision Sciences* 2010;41(2):373-401
5. Fralic M. Upskilling for new-era healthcare: Lessons from high impact readings. *Nurse Leader* 2013;11(4)
6. Rimmer A. Upskill doctors to make the most of new medical technologies, report says. *BMJ* 2018;361(k2861)

For peer review only

7. Almomani E, Sullivan J, Hajjieh M, et al. Simulation-based education programme for upskilling non-critical care nurses for COVID-19 deployment. *BMJ Simulation and Technology Enhanced Learning* 2020
8. NHS. Allied Health Professions. 2020. https://www.england.nhs.uk/ahp/.
9. Brown C, Wickline M, Ecoff L, et al. Nursing practice, knowledge, attitudes and perceived barriers to evidence-based practice at an academic medical centre. *Journal of Advanced Nursing* 2008;65:371–81
10. Dawes M, Summerskill W, Glasziou P, et al. Sicily statement on evidence-based practice. *BMC Medical Education* 2005;5(1):1-7
11. Malik G, McKenna L, Plummer V. Perceived knowledge, skills, attitude and contextual factors affecting evidence-based practice among nurse educators, clinical coaches and nurse specialists. *International Journal of Nursing Practice* 2014;21(S2):46-57
12. Gordon C, Thompson A. Use of personal protective equipment during the COVID-19 pandemic. *British Journal of Nursing* 2020;29(13)
13. Michalec B, Lamb G. COVID-19 and team-based healthcare: The essentiality of theory-driven research. *Journal of Interprofessional Care* 2020;34(5):593-99
14. Portnoy J, Waller M, Elliot T. Telemedicine in the Era of COVID-19. *The Journal of Allergy and Clinical Immunology: In Practice* 2020;8(5):1489-91
15. Wosik J, Fudim M, Cameron B, et al. Telehealth transformation: COVID-19 and the rise of virtual care. *Journal of the American Medical Informatics Association* 2020;27(6):957–62
16. Hatfield T, Withers T, Greaves C. Systematic review of the effect of training interventions on the skills of health professionals in promoting health behaviour, with meta-analysis of subsequent effects on patient health behaviours. *BMC Health Serv Res* 2020;20:593
17. Morris L, Horne M, McEvoy P, et al. Communication training interventions for family and professional carers of people living with dementia: a systematic review of effectiveness, acceptability and conceptual basis. *Aging Ment Health* 2018;22(7):863-80
18. Gerrish K, Clayton J. Promoting evidence-based practice: An organizational approach. *Journal of Nursing Management* 2004;12:114–23
19. Pravikoff D, Tanner A, Pierce S. Readiness of US nurses for evidence-based practice. *The American Journal of Nursing* 2005;105:40–51
20. Thompson C, McCaughan D, Cullum N, et al. Barriers to Evidence-based practice in primary care nursing: Why viewing decision-making as context is helpful. *Journal of Advanced Nursing* 2005;52:432–44
21. Wilberforce M, Abendstern M, Tucker S, et al. Support workers in community mental health teams for older people: roles, boundaries, supervision and training. *J. Adv. Nurs* 2017;73(7):1657-66
22. Curtis K, Brooks S. Digital health technology: Factors affecting implementation in nursing homes. *Nursing Older People* 2020;32(2):14-21
23. Shirey M. Lewin's theory of planned change as a strategic resource. *The Journal of Nursing Administration* 2013;43(92):69–72
24. Keyworth C, Hart J, Armitage C, et al. What maximizes the effectiveness and implementation of technology-based interventions to support healthcare professional practice? A systematic literature review. *BMC Medical Informatics and Decision Making* 2018;18(1):93
25. Austin M, Claassen J. Impact of organizational change on organizational culture. *Journal of Evidence-Informed Social Work* 2008;5(1-2):321–59
26. Udon S, Wagner J. Common Change Theories and Application to Different Nursing Situations. In: Wagner J, ed. Leadership and Influencing Change in Nursing. Canada: University of Regina Press, 2018.
27. Bowers B. Managing change by empowering staff. *Nursing Times* 2011;107(32-33):19–21
28. Spagnoletti C, Bui T, Fischer G, et al. Implementation and evaluation of a web-based communication skills learning tool for training internal medicine interns in patient-doctor communication. *Journal of Communication in Healthcare* 2009;2(2):159-72

For peer review only

29. Yukl G. *Leading in organizations*. 8 ed. Upper Saddle River, NJ: Pearson, 2013.
30. Pottle J. Virtual reality and the transformation of medical education. *Future Healthc J* 2019
31. Seropian M, Brown K, Gavilanes J, et al. Simulation: not just a manikin. *J Nurs Educ* 2004;43(4):164-9
32. Al-Elq A. Simulation-based medical teaching and learning. *Journal of Family & Community Medicine* 2010;17(1):35–40
33. Alamrani M, Alammar K, Alqahtani S, et al. Comparing the Effects of Simulation-Based and Traditional Teaching Methods on the Critical Thinking Abilities and Self-Confidence of Nursing Students. *J Nurs Res* 2018;26(3):152-57
34. Blanié A, Amorim M, Benhamou D. Comparative value of a simulation by gaming and a traditional teaching method to improve clinical reasoning skills necessary to detect patient deterioration: A randomized study in nursing students. *BMC Medical Education* 2020;20(53)
35. William A, Vidal V, John P. Instruction Versus Virtual Reality Simulation: A Comparative Study of Phlebotomy Training among Nursing Students in Kuwait. *Journal of Education and Practice* 2016;7:18-25
36. Maddry J, Varney S, Sessions D, et al. A comparison of simulation-based education versus lecture-based instruction for toxicology training in emergency medicine residents. *Journal of Medical Toxicology: Official Journal of the American College of Medical Toxicology* 2014;10(4):364–68
37. Ziv A, Ben-David S, Ziv M. Simulation based medical education: an opportunity to learn from errors. *Med Teach* 2005;27(3):193-9
38. Freina L, Ott M. Literature review on immersive virtual reality in education: State of the art and perspectives. The International Scientific Conference eLearning and Software for Education, 2015:133.
39. Rogers S. Virtual reality: The learning aid of the 21st century. Secondary Virtual reality: The learning aid of the 21st century 2019.
40. Global Augmented Reality I.G. Global Augmented Reality (AR) and Virtual Reality (VR) Market in Healthcare Market to Reach $11.14 Billion by 2025. 2019. https://www.prnewswire.com/news-releases/global-augmented-reality-ar-and-virtual-reality-vr-market-in-healthcare-market-to-reach-11-14-billion-by-2025--300831306.html.
41. Balian S, McGovern S, Abella B, et al. Feasibility of an augmented reality cardiopulmonary resuscitation training system for health care providers. *5* 2019;8:e02205
42. Bric J, Lumbard D, Frelich M, et al. Current state of virtual reality simulation in robotic surgery training: A review. *Surg Endosc* 2016;30:2169
43. Fleming M, Olsen D, Stathes H, et al. Virtual Reality Skills Training for Health Care Professionals in Alcohol Screening and Brief Intervention. *The Journal of the American Board of Family Medicine* 2009;22(4):387-98
44. Freeman K, Thompson S, Allely E, et al. A Virtual Reality Patient Simulation System for Teaching Emergency Response Skills to U.S. Navy Medical Providers. *Prehospital and Disaster Medicine* 2001;16(1):3-8
45. Harrington C, Kavanagh D, Quinlan J, et al. Development and evaluation of a trauma decision-making simulator in Oculus virtual reality. *Am J Surg* 2018;215:42-47
46. Scerbo M, Bliss J, Schmidt E, et al. The efficacy of a medical virtual realitysimulator for training phlebotomy. *Hum Factors* 2006;48(1):72–84
47. Makransky G, Terkildsen T, Mayer R. Adding immersive virtual reality to a science lab simulation causes more presence but less learning. *Learn. Instr* 2019;60:225–36
48. Makransky G, Andreasen N, Baceviciute S, et al. Immersive virtual reality increases liking but not learning with a science simulation and generative learning strategies promote learning in immersive virtual reality. *J. Educ. Psych* 2020
49. Vidal V, Ohaeri B, John P, et al. Virtual Reality and the Traditional Method for Phlebotomy Training Among College of Nursing Students in Kuwait: Implications for Nursing Education and Practice. *Journal of Infusion Nursing* 2013;36(5):349-55
50. Bowyer M, Pimentel E, Fellows J, et al. Teaching intravenous cannulation to medical students: comparative analysis of two simulators and two traditional educational approaches. *Studies in Health Technology and Informatics* 2005;111:57–63

For peer review only

51. Aïm F, Lonjon G, Hannouche D, et al. Effectiveness of Virtual Reality Training in Orthopaedic Surgery. *Arthroscopy: The Journal of Arthroscopic & Related Surgery* 2016;32(1):224-32
52. Rourke S. How does virtual reality simulation compare to simulated practice in the acquisition of clinical psychomotor skills for pre-registration student nurses? A systematic review. *International Journal of Nursing Studies* 2020;102:103466
53. FundamentalVR. 2020. https://www.fundamentalvr.com.
54. SentiAR. Adding a New Dimension to Clinical Practice. 2020. https://sentiar.com.
55. Augmedics. xVision the future of surgery is within sight. n.d. https://augmedics.com.
56. GLANTA. SureWash Hand Hygiene App. 2020. https://surewash.com/surewash-app-practice-hand-hygiene/.
57. Gasteiger N, Dowding D, Ali S, et al. Sticky apps, not sticky hands: A systematic review and content synthesis of hand hygiene mobile apps. *Journal of the American Medical Informatics Association* 2021. In Press
58. Singh R, Javaid M, Kataria R, et al. Significant applications of virtual reality for COVID-19 pandemic. *Diabetes & metabolic syndrome* 2020;14(4):661–64
59. Horlick K. Virtual reality helps simulation team tackle Covid-19. 2020. https://www.bartshealth.nhs.uk/news/virtual-reality-helps-simulation-team-tackle-covid19--8664.
60. Pawson R, Tilley N. *Realistic Evaluation*. London: SAGE Publications Ltd, 1997.
61. Jagosh J, Macaulay A, Pluye P, et al. Uncovering the benefits of participatory research: implications of a realist review for health research and practice. *Milbank Q* 2012;90:1–41
62. Wong G, Westhorp G, Pawson R, et al. *Realist synthesis. RAMESES training materials*. London: The RAMESES Project, 2013.
63. Randell R, Wright J, Alvarado N, et al. What supports and constrains the implementation of multifactorial falls risk assessment and tailored multifactorial falls prevention interventions in acute hospitals? Protocol for a realist review
64. Davidoff F, Dixon-Woods M, Leviton L, et al. Demystifying theory and its use in improvement. *BMJ Quality & Safety* 2015;24:228-38
65. Wong G, Greenhalgh T, Westhorp G, et al. RAMESES publication standards: realist syntheses. *BMC Med* 2013;11:21
66. Wiese A, Kilty C, Bergin C, et al. Protocol for a realist review of workplace learning in postgraduate medical education and training. *Systematic Reviews* 2017;6(10)
67. Randell R, Honey S, Alvarado N, et al. Embedding robotic surgery into routine practice and impacts on communication and decision making: a review of the experience of surgical teams. *Cognition, Technology & Work* 2016;18(2):423–37
68. Pawson R, Greenhalgh T, Harvey G, et al. Realist review – a new method of systematic review designed for complex policy interventions. *J Health Serv Res Policy* 2005;10(suppl 1):21-34
69. Schon D. *Educating the reflective practitioner: Toward a new design for teaching and learning in the professions*. San Francisco: Jossey-Bass, 1987.
70. Slotnick H. How doctors learn: The role of clinical problems across the Medical School-to-Practice Continuum. *Academic Medicine* 1996:28-34
71. Mukhalalati B, Taylor A. Adult Learning Theories in Context: A Quick Guide for Healthcare Professional Educators. *Journal of Medical Education and Curricular Development* 2019;6
72. Kolb D. *Experiential Learning as the Science of Learning and Development*. Englewood Cliffs, NJ: Prentice Hall, 1984.
73. Kang L, Brian S, Ricca B. Constructivism in pharmacy school. *Curr Pharm Teach Learn* 2010;2(126–130)
74. Greening T. Building the constructivist toolbox: An exploration of cognitive technologies. *Educational Technology* 1998;38(2):28-35
75. Jonassen D, Hernandez-Serrano J, Choi I. Integrating constructivism and learning technologies. In: Spector M, Anderson TM, eds. Integrated and holistic perspectives on learning, instruction and technology: Understanding complexity Netherlands: Kluwer Academic, 2000:103-27.
76. Chen C. Theoretical Bases for Using Virtual Reality in Education. Themes in Science and Technology Education. Klidarithmos Computer Books, 2009.
77. Rogers E. *Diffusion of innovations*. 4 ed. New York: Free Press, 1995.

For peer review only

78. Davis F. Perceived usefulness, perceived ease of use, and user acceptance of information technology. *Management Information Systems Quarterly* 1989;13(3):319-40
79. Venkatesh V, Morris M, Davis G, et al. User acceptance of information technology: Toward a unified view. *Management Information Systems Quarterly* 2003;27(3):425-78
80. Greenhalgh T, Wherton J, Papoutsi C, et al. Beyond Adoption: A New Framework for Theorizing and Evaluating Nonadoption, Abandonment, and Challenges to the Scale-Up, Spread, and Sustainability of Health and Care Technologies. *JMIR* 2017;19(11):e367
81. Damschroder L, Aron D, Keith R, et al. Fostering implementation of health services research findings into practice: a consolidated framework for advancing implementation science. *Implementation Science* 2009;4:50
82. May C, Cummings A, Girling M, et al. Using Normalization Process Theory in feasibility studies and process evaluations of complex healthcare interventions: a systematic review. *Implementation Science* 2018;13(80)
83. Wong G, Greenhalgh T, Pawson R. Internet-based medical education: a realist review of what works, for whom and in what circumstances. *BMC Med Education* 2010;10(12)
84. Pantelidis P, Chorti A, Papagiouvanni I, et al. Virtual and Augmented Reality in Medical Education. In: Tsoulfas G, ed. Medical and Surgical Education - Past, Present and Future, 2018.
85. Hu H-Z, Feng X-B, Shao Z-W, et al. Application and prospect of mixed reality technology in medical field. *Curr Med Sci* 2019;39(1):1-6
86. Moher D, Liberati A, Tetzlaff J, et al. Preferred reporting items for systematic reviews and meta-analyses: The PRISMA statement. *PLOS Medicine* 2009;6(7):e1000097
87. Kirkpatrick D. *The Four Levels of Evaluation: Measurement and Evaluation*. Alexandria: American Society for Training and Development, 2007.
88. Kislov R, Pope C, Martin G, et al. Harnessing the power of theorising in implementation science. *Implementation Science* 2019;14(1):103
89. Wong G. Data gathering in realist reviews: Looking for needles in haystacks. In: Emmel N, Greenhalgh J, Manzano A, Monaghan M, Dalkin S, eds. Doing realist research. London: Sage, 2018:131-45.
90. Hong Q, FÀBregues S, Bartlett G, et al. The Mixed Methods Appraisal Tool (MMAT) version 2018 for information professionals and researchers. *Education for Information* 2018;34(4):285-91
91. Pluye P, Gagnon M, Griffiths F, et al. A scoring system for appraising mixed methods research, and concomitantly appraising qualitative, quantitative and mixed methods primary studies in Mixed Studies Reviews. *International Journal of Nursing Studies* 2009;46(4):529–46
92. Pace R, Pluye P, Bartlett G, et al. Testing the reliability and efficiency of the pilot Mixed Methods Appraisal Tool (MMAT) for systematic mixed studies review. *International Journal of Nursing Studies* 2012;49(1):47-53
93. Pawson R. Digging for Nuggets: How 'Bad' Research Can Yield 'Good' Evidence. *International Journal of Social Research Methodology* 2006;9(2):127-42
94. Funnell S, Rogers P. *Purposeful program theory: effective use of theories of change and logic models*. San Francisco: John Wiley & Sons, 2011.
95. Berg R, Denison E. Methodological strengths and limitations of the realist synthesis approach to systematic reviews—a promising methodology for the Cochrane Collaboration? The 20th Cochrane Colloquium. Auckland, New Zealand: John Wiley & Sons, 2012.
96. Shams F, Wong J, Nikoo M, et al. Understanding eHealth Cognitive Behavioral Therapy Targeting Substance Use: Realist Review. *J Med Internet Res* 2021;23(1):e20557

FIGURE LEGENDS

For peer review only

1 Figure 1. Summary of the three steps and process that will be conducted. CMO stands for Context
2 Mechanism and Outcome.

For peer review only

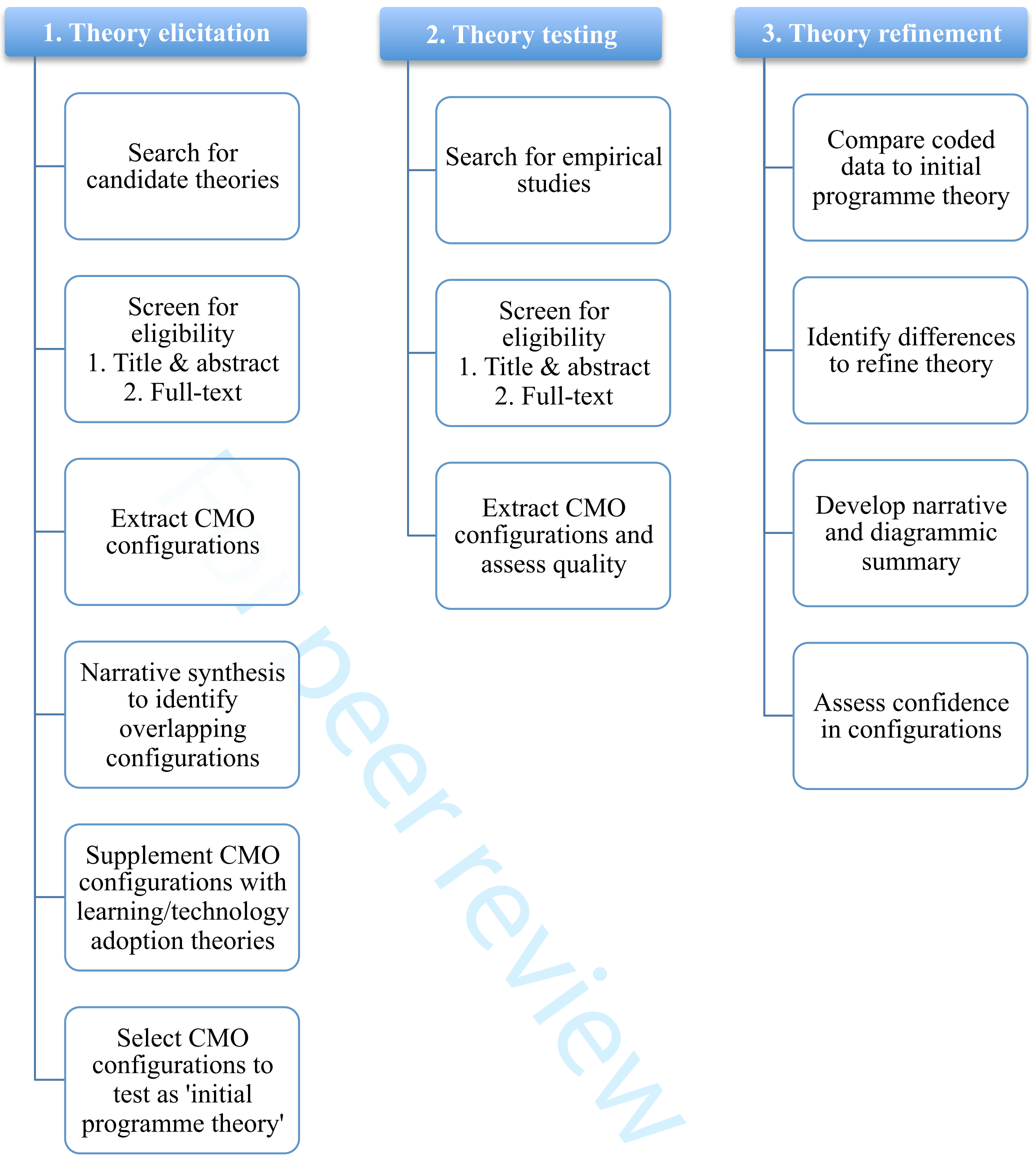

For peer review only

Supplementary file

Table S1. Completed checklist of the RAMESES Publication Standards for realist reviews[1]

**RAMESES* publication standards: realist syntheses**<br>*** Realist And MEta-narrative Evidence Syntheses: Evolving Standards**				
**No.**	**Section / Topic**	**Checklist item**	**Reported Yes**	**N/A**
**Title**				
**1.**		In the title, identify the document as a realist synthesis or review.	X	
**Abstract**				
**2.**		While acknowledging publication requirements and house style, abstracts should ideally contain brief details of: the study's background, review question or objectives; search strategy; methods of selection, appraisal, analysis and synthesis of sources; main results; and implications for practice	X	
**Introduction**				
**3.**	Rationale for review	Explain why the review is needed and what it is likely to contribute to existing understanding of the topic area.	X	
**4.**	Objectives and focus of review	State the objective(s) of the review and/or the review question(s). Define and provide a rationale for the focus of the review.	X	
**Methods**				
**5.**	Changes in the review process	Any changes made to the review process that was initially planned should be briefly described and justified.		X
**6.**	Rationale for using realist synthesis	Explain why realist synthesis was considered the most appropriate method to use.	X	
**7.**	Scoping the literature	Describe and justify the initial process of exploratory scoping of the literature.	X	
**8.**	Searching processes	While considering specific requirements of the journal or other publication outlet, state and provide a rationale for how the iterative searching was done. Provide details on all the sources accessed for information in the review. Where searching in electronic databases has taken place, the details should include, for example, name of database, search terms, dates of coverage and date last searched. If individuals familiar with the relevant literature and/or topic area were contacted, indicate how they were identified and selected.	X	
**9.**	Selection and appraisal of documents	Explain how judgements were made about including and excluding data from documents, and justify these.	X	
**10.**	Data extraction	Describe and explain which data or information were extracted from the included documents and justify this	X	

For peer review only

		selection.		
**11.**	Analysis and synthesis processes	Describe the analysis and synthesis processes in detail. This section should include information on the constructs analysed and describe the analytic process.	X	
**Results**				
**12.**	Document flow diagram	Provide details on the number of documents assessed for eligibility and included in the review with reasons for exclusion at each stage as well as an indication of their source of origin (for example, from searching databases, reference lists and so on). You may consider using the example templates (which are likely to need modification to suit the data) that are provided.		X
**13.**	Document characteristic s	Provide information on the characteristics of the documents included in the review.		X
**14.**	Main findings	Present the key findings with a specific focus on theory building and testing.		X
**Discussion**				
**15.**	Summary of findings	Summarise the main findings, taking into account the review's objective(s), research question(s), focus and intended audience(s).		X
**16.**	Strengths, limitations and future research directions	Discuss both the strengths of the review and its limitations. These should include (but need not be restricted to) (a) consideration of all the steps in the review process and (b) comment on the overall strength of evidence supporting the explanatory insights which emerged.<br>The limitations identified may point to areas where further work is needed.		X
**17.**	Comparison with existing literature	Where applicable, compare and contrast the review's findings with the existing literature (for example, other reviews) on the same topic.		X
**18.**	Conclusion and recommendat ions	List the main implications of the findings and place these in the context of other relevant literature. If appropriate, offer recommendations for policy and practice.		X
**19.**	Funding	Provide details of funding source (if any) for the review, the role played by the funder (if any) and any conflicts of interests of the reviewers.	X	

[1]Wong, G., Greenhalgh, T., Westhorp, G., et al. RAMESES publication standards: realist syntheses. *BMC Medicine* 2013;11(1):21.

For peer review only

Table S2. Initial search strategy

**Databases**	
	Medline<br>SCOPUS<br>CINAHL<br>EMBASE<br>Education Resource Information Centre<br>PsycINFO<br>Web of Science
**Keywords**	
Technology/Intervention	• augmented reality<br>• virtual reality
Population/sample	• health*<br>healthcare; health; health worker; health staff; health provider<br>• care*<br>care; carer; caregiving; caregiver; caring<br>• nurs*<br>nurse, nursing, nurses<br>• doctor<br>• surgeon
Focus	• training<br>• upskilling<br>• skill<br>• education<br>• evaluation<br>• implementation<br>• feasibility<br>• effectiveness
**Example with Boolean operators**	
	(TITLE-ABS-KEY (augmented AND reality OR virtual AND reality) AND TITLE-ABS-KEY (health* OR care* OR nurs* OR doctor OR surgeon) AND TITLE-ABS-KEY (training OR upskilling OR skill OR education) AND TITLE-ABS-KEY (evaluation OR implementation OR feasibility OR effectiveness))

For peer review only

Table S1. Completed checklist of the RAMESES Publication Standards for realist reviews[1]

RAMESES* publication standards: realist syntheses<br>* Realist And MEta-narrative Evidence Syntheses: Evolving Standards				
**No.**	**Section / Topic**	**Checklist item**	**Reported Yes**	**N/A**
**Title**				
**1.**		In the title, identify the document as a realist synthesis or review.	X	
**Abstract**				
**2.**		While acknowledging publication requirements and house style, abstracts should ideally contain brief details of: the study's background, review question or objectives; search strategy; methods of selection, appraisal, analysis and synthesis of sources; main results; and implications for practice	X	
**Introduction**				
**3.**	Rationale for review	Explain why the review is needed and what it is likely to contribute to existing understanding of the topic area.	X	
**4.**	Objectives and focus of review	State the objective(s) of the review and/or the review question(s). Define and provide a rationale for the focus of the review.	X	
**Methods**				
**5.**	Changes in the review process	Any changes made to the review process that was initially planned should be briefly described and justified.		X
**6.**	Rationale for using realist synthesis	Explain why realist synthesis was considered the most appropriate method to use.	X	
**7.**	Scoping the literature	Describe and justify the initial process of exploratory scoping of the literature.	X	
**8.**	Searching processes	While considering specific requirements of the journal or other publication outlet, state and provide a rationale for how the iterative searching was done. Provide details on all the sources accessed for information in the review. Where searching in electronic databases has taken place, the details should include, for example, name of database, search terms, dates of coverage and date last searched. If individuals familiar with the relevant literature and/or topic area were contacted, indicate how they were identified and selected.	X	
**9.**	Selection and appraisal of documents	Explain how judgements were made about including and excluding data from documents, and justify these.	X	
**10.**	Data extraction	Describe and explain which data or information were extracted from the included documents and justify this selection.	X	

For peer review only - http://bmjopen.bmj.com/site/about/guidelines.xhtml

**11.**	Analysis and synthesis processes	Describe the analysis and synthesis processes in detail. This section should include information on the constructs analysed and describe the analytic process.	X	
**Results**				
**12.**	Document flow diagram	Provide details on the number of documents assessed for eligibility and included in the review with reasons for exclusion at each stage as well as an indication of their source of origin (for example, from searching databases, reference lists and so on). You may consider using the example templates (which are likely to need modification to suit the data) that are provided.		X
**13.**	Document characteristic s	Provide information on the characteristics of the documents included in the review.		X
**14.**	Main findings	Present the key findings with a specific focus on theory building and testing.		X
**Discussion**				
**15.**	Summary of findings	Summarise the main findings, taking into account the review's objective(s), research question(s), focus and intended audience(s).		X
**16.**	Strengths, limitations and future research directions	Discuss both the strengths of the review and its limitations. These should include (but need not be restricted to) (a) consideration of all the steps in the review process and (b) comment on the overall strength of evidence supporting the explanatory insights which emerged.<br>The limitations identified may point to areas where further work is needed.		X
**17.**	Comparison with existing literature	Where applicable, compare and contrast the review's findings with the existing literature (for example, other reviews) on the same topic.		X
**18.**	Conclusion and recommendat ions	List the main implications of the findings and place these in the context of other relevant literature. If appropriate, offer recommendations for policy and practice.		X
**19.**	Funding	Provide details of funding source (if any) for the review, the role played by the funder (if any) and any conflicts of interests of the reviewers.	X	

[1]Wong, G., Greenhalgh, T., Westhorp, G., et al. RAMESES publication standards: realist syntheses. *BMC Medicine* 2013;11(1):21.

For peer review only
